# Outcome and Complications of MR Guided Focused Ultrasound for Essential Tremor: A Systematic Review and Meta-Analysis

**DOI:** 10.3389/fneur.2021.654711

**Published:** 2021-05-07

**Authors:** Mohit Agrawal, Kanwaljeet Garg, Raghu Samala, Roopa Rajan, Vikas Naik, Manmohan Singh

**Affiliations:** ^1^Department of Neurosurgery, All India Institute of Medical Sciences, Jodhpur, India; ^2^Department of Neurosurgery, All India Institute of Medical Sciences, New Delhi, India; ^3^Department of Neurology, All India Institute of Medical Sciences, New Delhi, India; ^4^Department of Neurosurgery, Bangalore Medical College, Bangalore, India

**Keywords:** cerebellothalamic tract, diffusion tensor imaging, essential tremor, magnetic resonance guided focused ultrasound, targeting technique comparison, ventral intermediate nucleus

## Abstract

**Background:** Magnetic resonance guided focused ultrasound (MRgFUS) is a relatively novel technique to treat essential tremor (ET). The objective of this review was to analyze the efficacy and the safety profile of MRgFUS for ET.

**Methods:** A systematic literature review was done. The post procedure changes in the Clinical Rating Scale for Tremor (CRST) score, hand score, disability and quality of life scores were analyzed.

**Results:** We found 29 studies evaluating 617 patients. DTI based targeting was utilized in six cohorts. A significant difference was observed in the pooled standard mean difference between the pre and postoperative total CRST score (*p*-value < 0.001 and 0.0002), hand score (*p*-value 0.03 and 0.02); and the disability at 12 months (*p*-value 0.01). Head pain and dizziness were the most in procedure complications. The immediate pooled proportion of ataxia was 50%, while it was 20% for sensory complications, which, respectively, declined to 31 and 13% on long term follow up. A significant reduction (*p* = 0.03) in immediate ataxia related complications was seen with DTI targeting.

**Conclusion:** MRgFUS for ET seems to be an effective procedure for relieving unilateral tremor. Use of DTI based targeting revealed a significant reduction in post procedure ataxia related complications as compared to traditional targeting techniques. Analysis of other complications further revealed a decreasing trend on follow up.

## Introduction

Essential tremor (ET) is the most common form of adult movement disorder (1, 2), with an estimated prevalence of 4–6% (3, 4). Although not life threatening, it carries significant morbidity due to functional impairment from loss of hand function (5). Medications such as propranolol and primidone are the first line therapy, but many patients with ET become drug refractory (5). These patients can be good candidates for surgical treatments such as deep brain stimulation, radiofrequency thalamotomy, focused ultrasound thalamotomy, or gamma knife thalamotomy (GKT).

Stereotactic radiofrequency ablation of basal ganglia and thalamic structures, including the VIM nucleus was one of the first surgical interventions to be offered to ET patients (6). The higher risk of side effects eventually led clinicians to consider DBS as a choice for surgical treatment of ET (7–9). However, there are several drawbacks related to its use, such as implant related complications and the requirement of frequent hospital visits for programming (10, 11). GKT was developed as a relatively less invasive thalamotomy method, but suffered from unpredictable lesion size limitation and time taken for the clinical benefit to become apparent (12). MRgFUS integrates ultrasonic waves with magnetic resonance imaging for therapeutic transcranial ablation (13). MRgFUS thalamotomy is an image guided procedure with no incision. It is a precision thalamotomy, in other words. Advantages of MRgFUS include non-invasiveness, real time real-time visualization of the thermal spot, and temperature monitoring while testing for a clinical response during lesion creation. Moreover, there are no hardware-related complications, and the patients do not require repeated hospital visits for programming. New advances are being made to improve the results of MRgFUS. Diffusion tensor imaging, which allows the delineation of the CTT, has been incorporated in recent times in an effort to improve the target of the ultrasonic waves (14).

An earlier review published on the topic included only nine studies (15). Many errors were pointed out in the article, including an insufficient number of studies to draw relevant conclusions (16). Additionally, no long-term data were available at that time. Since then, several centers around the world have embraced this technique. This review summarizes the latest available evidence in literature in terms of efficacy and complications of MRgFUS for ET. Owing to the paucity of Class I evidence and the unlikelihood of prospective studies comparing the various surgical techniques available for treating ET, this meta-analysis strives to provide pooled results of a number of smaller studies on the topic.

## Methods

### Literature Search

**Figure 1:**
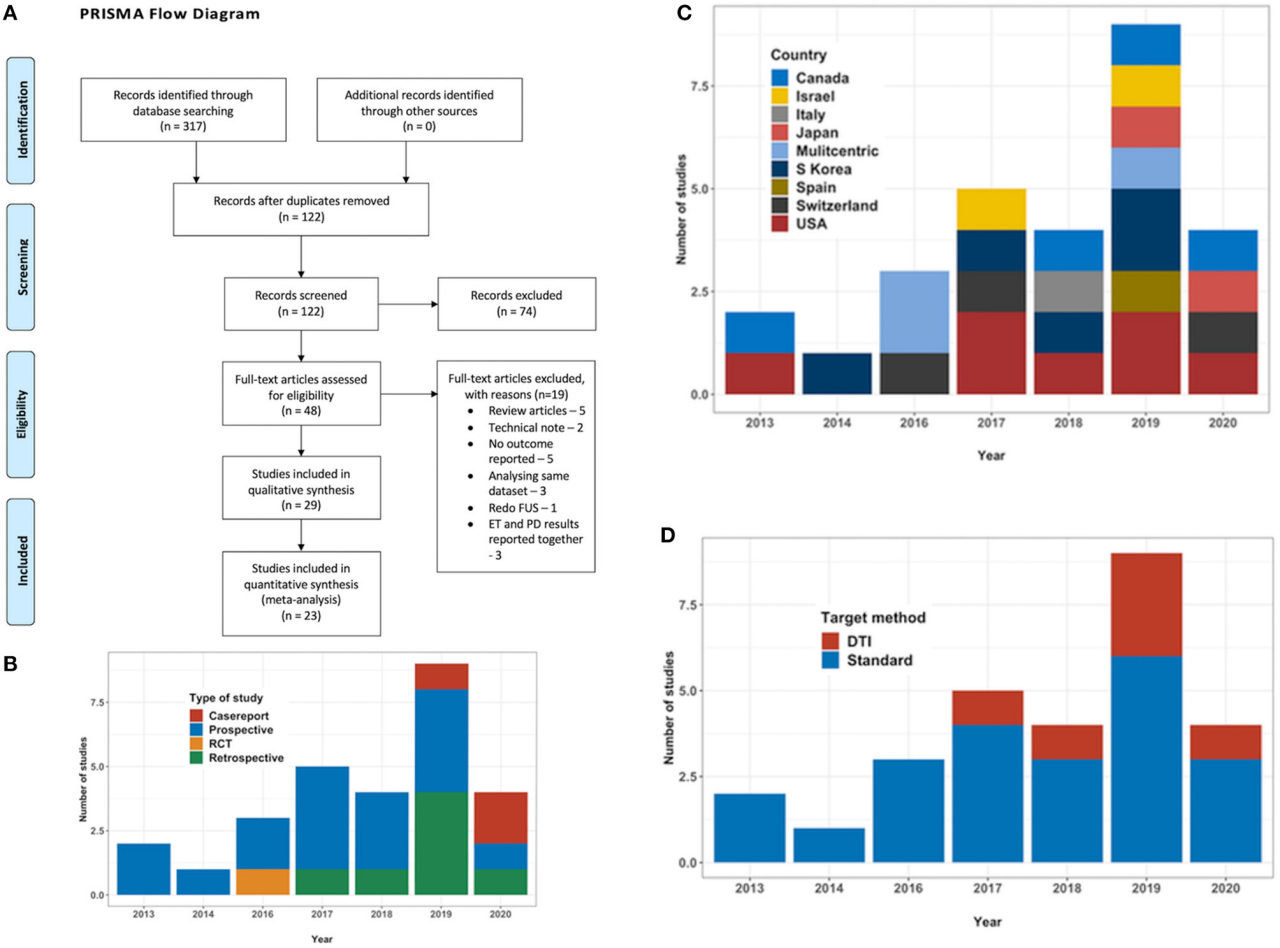
**(A)** PRISMA flow diagram outlining the literature search process. **(B)** Bar chart depicting the type and number of studies with year of publication. **(C)** Bar chart depicting the country where the study was conducted. **(D)** Bar chart depicting the trend in targeting technique over the years.

A search for published literature till May 2020 was done on PubMed, Google Scholar, Cochrane library database and Medline using the keywords “MR guided,” “focused ultrasound,” “essential tremor,” “thalamotomy,” “ventral intermediate nucleus,” “cerebellothalamic,” and “diffusion imaging” in various combinations. References of the relevant studies and other review articles on the subject were also studied to supplement the initial search. Only English language articles were considered. Two authors manually and independently reviewed all publications encountered during the search. Disagreements, if any, were resolved with the opinion of a third independent observer. PRISMA guidelines were followed throughout (Figure 1A).

### Inclusion and Exclusion Criteria

Studies describing the use of MRgFUS for the treatment of medically refractory ET (unilateral or bilateral) in the adult population were selected for this review. The exclusion criteria were studies that reported outcomes on patients with tremors secondary to any other causes, such as drug-induced tremor, history of preceding trauma within 3 months, psychogenic tremor, or co-morbid Parkinson disease and dystonia were excluded. For studies with mixed diagnoses, we only included outcomes reported for ET patients. To keep the focus primarily on MRgFUS, we excluded cases where a previous procedure such as DBS, radiosurgery or stereotactic ablation was done. To avoid duplication of results, we only included outcomes from a single publication where multiple publications reported outcomes from the same study cohort.

### Data Extraction

Data were extracted by two authors independently. Clinical data collected included the maximum reported period of follow up, the total Clinical Rating Scale for Tremor score (maximum score 160) (17, 18), hand score – a subset of CRST Part A and B (maximum score 32), disability as CRST Part C (maximum score 32) and quality of life as determined by the Quality of Life in Essential Tremor Questionnaire score (0–100%). All the data points were collected using a standardized data collection instrument developed in Microsoft Excel (Microsoft Inc., Redmond, WA) template.

The *primary outcome* variable was the change in CRST score pre and postoperatively at 3 and 12 months. The *secondary outcomes* were the difference in disability and QOL scores. For the meta-analysis, studies that reported the outcome as a mean value (with standard deviation) of the total CRST score, hand score, CRST Part C score and QUEST score were included. Studies reporting outcome as median or percentage improvement in outcome scores, studies that reported the hand score out of 12/16 were excluded from that part of the analysis. Case reports were excluded from the meta-analysis.

Complications attributed to the procedure were recorded as immediate (occurring during the procedure to within 48 h after the procedure), short-term (from the third day onwards till 3 months), and long-term (persisting/appearing more than 3 months later). The complications were divided into two broad categories - neurologic and minor/treatment related. The neurological complications were further divided into four subcategories – sensory (paresthesia, taste disturbance, dysesthesia, tinnitus), motor (facial or limb weakness), ataxia (dizziness, gait ataxia, dysmetria/hand ataxia) and speech & swallowing. The minor/treatment related complications were categorized as headache and fatigue, sonication, MRI, frame related and others.

### Statistical Analysis

Statistical analysis of the pooled data was performed using R software (R Foundation for Statistical Computing, Vienna, Austria) employing the “meta” and “metaphor” packages (19–21). We first performed the analysis using fixed-effect modeling and later, with random-effect methods (after assessing heterogeneity with fixed modeling). Thus, all values reported in the current analysis were from random-effect modeling (was heterogeneity significant for all analyses). The extent of heterogeneity between the studies was quantified using the *I*^2^ statistic. Values of *I*^2^ < 25%, 25–75%, and >75% were defined as low, moderate and high heterogeneity, respectively (22). The results were expressed as a standardized mean difference with a 95% confidence interval. A negative SMD indicates improvement in the relevant score postoperatively. *P*-value < 0.05 was considered as statistically significant. Linear regression analysis was performed to detect any significant correlation between parameters.

### Risk of Bias Assessment

Studies were assessed for a possible publication bias initially using a funnel plot, which was later quantified using Egger's test. Publication bias was evaluated for reporting of CRST total score at 3 months. Egger's regression test showed that the X-axis intercept occurred at −1.587 with *p*-value (two-tailed) being 0.04315 (Supplementary Material 1).

### Study Quality Assessment

The MINORS criteria were used to assess the methodological quality of non-randomized surgical studies (23) (Table 1).

**Table 1:** Methodological index for non-randomized studies (MINORS) scores for included studies^*^.

**References**	**Clearly stated aim**	**Inclusion of consecutive patients**	**Prospective collection of data**	**Endpoints appropriate to aim of study**	**Unbiased assessment of the study endpoint**	**Follow up period appropriate to aim of study**	**Loss to follow up < 5%**	**Prospective calculation of study size**	**Total score**
Lipsman et al. (24)	2	2	2	2	1	1	2	0	12
Elias et al. (25)	2	2	2	2	1	1	2	0	12
Chang et al. (26)	2	2	2	2	1	1	2	0	12
Gallay et al. (27)	2	2	2	2	1	1	1	0	11
Zaroor et al. (28)	2	2	2	2	1	1	2	0	12
Schreglmann et al. (29)	2	2	2	2	2	1	2	0	13
Kim et al. (30)	2	2	0	2	1	1	2	0	10
Chazen et al. (31)	2	2	2	2	1	0	0	0	9
Federau et al. (32)	2	2	0	2	1	1	2	0	9
Jung et al. (33)	2	2	2	2	1	1	2	0	12
Iacopino et al. (34)	2	2	2	2	1	1	1	0	11
Krishna et al. (35)	2	2	2	2	2	1	2	0	13
Boutet et al. (36)	2	2	0	2	1	1	2	0	10
Park et al. (37)	2	2	2	2	2	2	1	0	13
Hori et al. (38)	2	2	0	2	1	1	2	0	10
Pineda-Pardo et al. (39)	2	1	2	2	1	1	1	0	10
Jones et al. (40)	2	2	0	2	2	1	1	0	10
Sinai et al. (41)	2	2	2	2	1	1	1	0	11
Chang et al. (42)	2	2	1	2	1	1	2	0	11
Miller et al. (43)	2	2	0	2	1	1	1	0	8
Krishna et al. (44)	2	2	0	2	1	1	2	0	10
Gallay et al. (45)	2	2	2	2	1	1	2	0	12
Fukutome et al. (46)	2	2	0	2	1	1	2	0	10

* *Score per criterion: 0, not reported; 1, reported but inadequate; 2, reported and adequate. Ideal global score for non-comparative study is 16*.

## Results

A total of 29 studies (24–52), evaluating a total of 617 patients (156 female) fulfilled the inclusion criteria for the systematic review. Out of these 29 studies, there was only one RCT (47), with the rest being observational studies. There were fourteen prospective and eight retrospective studies and three case reports (Figure 1B). At present, the procedure has been performed in eight countries (Figure 1C). Three studies were reporting long term follow-up (37, 48, 49) of the patients in the RCT. Mean age of the patients ranged from mean 61.7 ± 8.1 to 78 ± 6 years in the studies, except a case report which reported the use of MRgFUS in treating nonagenarians (51). Mean disease duration ranged from 15.4 ± 13.3 to 34.3 ± 22.1 years. The maximum follow-up reported was 5 years by Sinai et al. in two patients (41). The baseline characteristics of the studies are summarized in Table 2.

**Table 2:** Study details arranged chronologically by the month and year of publication along with follow up and outcome scores [^*^For descriptive purposes, the cohort of patients reported by Krishna et al. (35) and Elias et al. (47) were split into two groups, each with its own distinct characteristics].

**References**	**Study design**	**Period of recruitment of patients**	**Place where conducted**	**No. of patients**	**Mean age ± SD (range) (in years)**	**Sex (male, female)**	**Mean disease duration ± SD (range) (in years)**	**Target**	**Localization method**	**Maximum follow up (range)**	**Mean total CRST score**		**CRST part A**		**Hand score**		**CRST part C**		**QUEST**	
											**Preoperative**	**Postoperative (follow up – number of patients)**	**Preoperative**	**Postoperative (follow up – number of patients)**	**Preoperative (maximum score)**	**Postoperative (follow up – number of patients)**	**Preoperative**	**Postoperative (follow up – number of patients)**	**Preoperative**	**Postoperative (follow up – number of patients)**
Lipsman et al. (24)	Prospective, uncontrolled	May 2012– January 2013	Toronto, Canada	4	70.8 ± 7.8 (58–77)	4, 0	17.8 ± 8.2 (6–25)	VIM	Standard	3 month	70.75 ± 17.0	35.25 ± 9.5	NA	NA	7.25 ± 1.9 (out of 12)	1.25 ± 0.82 (3 m)	20.75 ± 3.9	10.25 ± 2.8	NA	NA
Elias et al. (25)	Prospective, uncontrolled	February–December 2011	Virginia, USA	15	66.6 ± 8.0 (53 to 79)	10, 5	32.0 ± 21.3 (4–60)	VIM	Standard	1 year	54.9 ± 14.4	24.3 ± 14.8	NA	NA	20.4 ± 5.2 (out of 32)	4.3 ± 3.5 (3 m), 5.2 ± 4.8 (1 yr)	18.2 ± 4.1	2.8 ± 3.4	NA	NA
Chang et al. (26)	Prospective, uncontrolled	March–November 2012	Seoul, Korea	8	66.1 ± 5.3 (61–78)	7, 1	32.1 ± 16.1 (15–57)	VIM	Standard	6 month	NA	NA	5.1 ± 1.2	1.4 ± 1.4	NA	NA	13.5 ± 3.7	2.8 ± 2.8	NA	NA
Gallay et al. (27)	Prospective, uncontrolled	NA	Solothurn and Bern, Switzerland	21	69.1 ± 9.2	15, 6	29.9 ± 15	CTT	Standard	1 year	57.6 ± 13.2	25.8 ± 17.6 (1 yr−10)	NA	NA	NA	NA	NA	NA	NA	NA
Elias et al. (47) (Treatment group)^*^	Randomized Control Trial	August 2013–September 2014	Multicentric-8	56	70.8 ± 8.7	37, 19	28.3 ± 16.4	VIM	Standard	1 year	50.1 ± 14.0	29.6 ± 13 (3 m); 32.4 ± 14.5 (12 m)	NA	NA	18.1 ± 4.8 (out of 32)	9.6 ± 5.1 (3 m), 10.9 ± 4.5 (1 yr)	16.5 ± 4.6	6.2 ± 5.6 (3 m), 6.3 ± 6.2 (1 yr)	42.6 ± 18.3	23.1 ± 16.9 (3 m), 41.4 ± 19.4 (1 yr)
Elias et al. (47) (Sham Crossover)^*^	Randomized Control Trial	August 2013–September 2014	Multicentric-8	21 (19 crossover, 2 retreat)	71.4 ± 7.3	15, 5	27.9 ± 14.9	VIM	Standard	1 year	45.43 ± 12.55	23.48 ± 10.95 (3 m); 25.00 ± 11.11 (6 m); 18.67 ± 16.02 (1 yr−9)	NA	NA	16.5 ± 4.21 (out of 32)	7.43 ± 3.88 (3 m),8.00 ± 3.86 (6 m), 6.71 ± 4.7 (1 yr−9)	NA	NA	NA	NA
Chang et al. (48)	2 year follow up of patients in RCT by Elias et al. (47)	August 2013–September 2014	Multicentric-8	76 (67 followed till 2 years)	71.0 ± 8.3 (47–89)	52, 24	16.8 ± 12.3	VIM	Standard	2 year	NA	NA	NA	NA	19.8 ± 4.9 (out of 32)	8.9 ± 4.8 (1 yr−70), 8.8 ± 5.0 (2 yr−67)	16.4 ± 4.5	5.4 ± 5.3 (1 yr −70) 6.5 ± 5.0 (2 yr−67)	NA	NA
Halpern et al. (49)	3 year follow up of patients in RCT by Elias et al. (47)	August 2013–September 2014	Multicentric-8	76 (52 followed till 3 years)	71.0 ± 8.3 (47–89)	52, 24	16.8 ± 12.3	VIM	Standard	3 year	NA	NA	NA	NA	20.1 ± 4.7 (out of 32)	9.5 ± 5.4	16.4 ± 4.5	7.5 ± 6.1	43.1 ± 18.3	23.8 ± 19.6
Zaroor et al. (28)	Prospective, uncontrolled	November 2013–January 2016	Haifa, Israel	18	73.1 ± 6.2 (64–87)	12, 6	15.5 ± 9.3 (5–30)	VIM	Standard	12.5 ± 7.0 (3–24) month	40.7 ± 11.6	9.3 ± 7.1 (1 m); 8.2 ± 5.0 (6 m)	NA	NA	NA	NA	NA	NA	44.8 ± 12.9	13.1 ± 13.2 (1 m); 12.3 ± 7.2 (6 m)
Schreglmann et al. (29)	Prospective, uncontrolled	NA	St. Gallen, Switzerland	6	70.7 ± 8.5 (58–82)	2, 4	24.5 ± 22.5 (2–56)	CTT	Standard	6 month	43.8 ± 9.8	19.8 ± 6.8	NA	NA	14.3 ± 4.9 (out of 32)	2.5 ± 2.6	NA	NA	NA	52% improvement
Kim et al. (30)	Retrospective	2012–2014	Seoul, South Korea	23	64.7 (47–77)	20, 3	20.5 (5–54)	VIM	Standard	1 year	NA	NA	NA	NA	NA	(>90% improvement was taken as success) 21 patients (91.3%) at 1 m, 18 (78.3%) at 12 m	NA	NA	NA	NA
Chazen et al. (31)	Prospective, uncontrolled	NA	New York, USA	4	64.25 ± 11.7	3, 1	NA	CTT	DTI based	NA	NA	NA	NA	NA	3.75 ± 1.0 (out of 15)	0.25 ± 0.50 (Immediate post treatment)	NA	NA	NA	NA
Federau et al. (32)	Retrospective	August 2013–May 2014	Stanford, USA	7	78 ± 6	5, 2	NA	VIM	Standard	1 year	NA	NA	6.5 ± 1.3	2.3 ± 1.1	21.5 ± 2.0 (out of 32)	9.7 ± 5.2	NA	NA	NA	NA
Jung et al. (33)	Prospective, uncontrolled	March 2012–September 2014	Seoul, South Korea	20	64.1 (47–77)	17, 3	21.2 (5–54)	VIM	Standard	1 year	44.75 ± 9.57	14.65 ± 9.19	12.60 ± 3.80	2.75 ± 3.18	18.15 ± 3.96 (out of 32)	5.80 ± 4.53	12.80 ± 3.17	5.75 ± 4.25	64.16 ± 17.75	27.38 ± 13.96
Iacopino et al. (34)	Prospective, uncontrolled	January 2015–September 2017	Palermo, Italy	13	65.22 ± 11.87	10, 3	22.38 (3–70)	VIM	Standard	6 month	40.2 ± 11.8	17.3 ± 7.31 (3 m); 17.7 ± 8.80 (11 pts−6 m)	NA	NA	6.4 ± 2.97 (out of 16)	2.1 (3 m), 2.2 (6 m−11)	NA	NA	35.09± 12.25	17.09 ± 10.67(3 m), 18.44 ± 13.76 (6 m−11)
Krishna et al. (35)	Prospective, uncontrolled	July 2015–September 2016	Ohio, USA	10	70.8 ± 9.7	6, 4	34.3 ± 22.1	VIM	DTI based	6 month	59.3 ± 17.3	29 ± 16 (3 m), 32 ± 15.9 (6 m−9)	20.7 ± 8	11.6 ± 6.5 (3 m)	17.4 ± 4.5 (out of 32)	6.5 ± 3.7 (3 m)	18.1 ± 5.1	4.3 ± 4.4 (3 m)	81.7 ± 17.7	45.3 ± 11.6 (3 m), 45.6 ± 10.8 (6 m−9)
Boutet et al. (36)	Retrospective	May 2012–August 2017	Toronto, Canada	66	72.4 ± 8.4	47, 19	23.0 ± 14.4	VIM	NA	3 month	59.7 ± 17.4	34.8 ± 14.4	NA	NA	NA	NA	NA	NA	NA	NA
Park et al. (37)	Prospective, uncontrolled [4 year follow up of patients reported in RCT by Elias et al. (47)]	October 2013–August 2014	Seoul, South Korea	12	61.7 ± 8.1 (47–72)	10, 2	17.8 ± 13.03 (7–54)	VIM	Standard	4 year	NA	NA	NA	NA	17.4 ± 3.8 (out of 32)	5.3 ± 3.4 (1 yr), 6.9 ± 5.9 (2 yr), 7.5 ± 5.3 (3 yr), 7.7 ± 4.1 (4 yr)	12.7 ± 3.0	2.9 ± 2.4 (1 yr), 5.1 ± 3.6 (2 yr), 4.4 ± 3.3 (3 yr), 4.7 ± 3.0 (4 yr)	NA	NA
Hori et al. (38)	Retrospective	April 2015–October 2017	Tokyo, Japan	12	76.5 ± 3.8 (67–82)	9, 3	Median 15 (10–70)	VIM	Standard	1 year	NA	NA	NA	NA	NA	NA	NA	NA	NA	NA
Pineda-Pardo et al. (39)	Prospective, uncontrolled	NA	Madrid, Spain	24	68.0 ± 10.1	17, 7	18.6 ± 12.8	VIM + CTT	Atlas + DTI based (to extend the target)	1 year	52.9 ± 13.0	23.8 ± 8.3 (3 m); 26.4 ± 11.3 (1 yr−19)	5.6 ± 1.8	1.0 ± 0.9 (3 m), 1.5 ± 1.3 (1 yr−19)	NA	NA	17.3 ± 4.8	4.2 ± 4.1 (0–15) (3 m), 5.4 ± 4.9 (0–19) (1 yr−19)	NA	NA
Yang et al. (50)	Case Report	NA	Philadelphia, USA	1	74	1, 0	1	CTT	DTI based	3 month	25	5 (3 m)	NA	NA	10 (out of 32)	1 (3 m)	9	0 (3 m)	NA	NA
Jones et al. (40)	Retrospective	July 2015–July 2018	Toronto, Canada	19 low temperature (LT), 30 high temperature (HT)	NA	NA	NA	VIM	Standard	1 year	NA	NA	NA	NA	20.5 ± 5.8 (Low Temperature - LT), 20.3 ± 5.0 (Hight Temperature - HT) (out of 32)	Improvement by 53% ± 32 and 51% ± 22% at 3 m, 45% ± 55% and 44% ± 22% (1 yr−9 LT, 27 HT)	NA	NA	NA	NA
Sinai et al. (41)	Prospective, uncontrolled	Nov 2013–Nov 2018	Haifa, Israel	44	Median 70.5 (63–87)	27, 17	16.3 ± 10.4 (1–30)	VIM	Standard	Median 12 month	Median 46.0	Median 12.0 (1 m−44); 18.0 (1 yr−24); 11.0 (2 yr−15); 16.0 (3 yr−10); 14.0 (4 yr−6); 8.0 (5 yr−2)	NA	NA	Median 19 (out of 32)	Median 0.0 (1 m−44); 4.0 (1 yr−24); 4.0 (2 yr−15); 3.5 (3 yr−10); 5.0 (4 yr−6); 3.0 (5 yr−2)	NA	NA	41.5	Median 5.5 (1 m−44); 14.0 (0–89) (1 yr−24); 15.0 (2 yr−15); 15.5 (3 yr−10); 14.5 (4 yr−6); 11.0 (5 yr−2)
Chang et al. (42)	Prospective, uncontrolled	since 2013	Seoul, South Korea	50	66.65 ± 9.95 (45–80)	42, 8	NA	VIM	Standard	17.8 ± 19.8 (1–60) month	NA	NA	NA	NA	12.12 ± 0.51 (out of 32)	5.88 ± 0.52	12.52 ± 0.52	3.64 ± 0.47	NA	NA
Miller et al. (43)	Retrospective	July 2014–August 2016	Baltimore, USA	4	NA	NA	NA	VIM + CTT	Atlas + DTI based (to extend the target)	3 month (1 patient died of unrelated cause after 3 months. For the rest, benefit was sustained till 1 year follow up, no scores mentioned)	57.5 ± 16.8	29.5 ± 6.4	NA	NA	6.5 ± 1.0 (out of 16)	0.75 ± 0.9	NA	NA	NA	NA
Krishna et al. (44) (Pivotal)^*^	Retrospective	2013–2015	Multicentric - 8	75 (treatment + sham crossover)	71.3 ± 8.4	51, 24	16.8 ± 12.3	VIM	Standard	1 year	NA	NA	NA	NA	19.9 ± 5 (out of 32)	Improvement: 56.3 ± 25.5% (3 m), 52.1 ± 24.9% (1 yr)	NA	Improvement : 68.3 ± 27.6% (3 m), 65.9 ± 30.9% (1 yr)	NA	NA
Krishna et al. (44) (Post Pivotal)^*^	Retrospective	2015–2016	Multicentric - 18	114	71 ± 9.5	80, 34	15.4 ± 13.3	VIM	Standard	1 year	NA	NA	NA	NA	19.3 ± 5 (out of 32)	Improvement: 63.6 ± 26.1% (3 m), 61.9 ± 24.9% (1 yr)	NA	Improvement: 72.3 ± 25.9% (3 m), 66.1 ± 32.1% (1 yr)	NA	NA
Gallay et al. (45)	Prospective, uncontrolled	After 2016	Solothurn & Bern, Switzerland	10	66 ± 8 years	8, 2	31 ± 14	CTT (3 patients also had a contralateral centrum medianum thalamotomy)	Standard	1 year	48 ± 12	16 ± 7 (3 m); 17 ± 8 (1 yr)	11.8 ± 3.9	3.6 ± 1.5 (3 m), 4.3 ± 1.9 (1 yr)	NA	NA	14.2± 3.4	2.6 ± 2.0 (3 m), 3.4 ± 2.6 (1 yr)	NA	NA
Paff et al. (51)	Case Report	NA	Toronto, Canada	1	93	1, 0	40	VIM	Standard	1 year	NA	52% improvement	NA	NA	NA	64% improvement in hand score	NA	NA	NA	NA
Buch et al. (52)	Case Report	NA	Philadephia, USA	1	80	1, 0	NA	VIM + CTT	DTI based	6 week	NA	NA	NA	NA	20 (out of 32)	2	21	2	NA	NA
Fukutome et al. (46)	Retrospective	May 2016–August 2017	Nara, Japan	15	62.9 ± 11.3 (41–82)	11, 4	21.5 ± 14.0 (2–47)	VIM	Standard	1 year	NA	NA	NA	NA	18.5 ± 5.8 (out of 32)	4.6 ± 5.7	NA	NA	NA	NA

*RCT, randomized controlled trial; SD, standard deviation; VIM, ventral intermediate nucleus; CTT, cerebello-thalamic tract; DTI, diffusion tensor imaging; NA, not available; m, month; yr, year*.

### Targeting Method and Operative Parameters

Majority of the studies followed atlas-based targeting which was further refined by direct targeting based on MRI. DTI based targeting was reported by six studies, two of whom were case reports (Table 2, Figure 1D). Treatment parameters used by various centers have been summarized in Table 3. The skull density ratio (SDR) was more than a mean of 0.45 for all studies, except one which reported a median value of 0.38 (38). The mean number of sonications ranged from 11 ± 3.2 to 22.5 ± 7.6. All studies reported maximum temperature attained as >55°C for the lesioning except Chang et al. (26), who reported 53 ± 3.3°C as the mean temperature attained, and Jones et al. (40), who described a series of 19 patients in whom multiple low-temperature sonications were used to create a lesion. The maximum energy delivered ranged from a mean of 10,320 ± 4,537 to 16,910 ± 8,340 J. The sonication time ranged from a mean of 82.8 ± 30.8 to 105 ± 55 min. A recent case report mentioned 80 min as the sonication time (51). Four centers utilized a 1.5T MRI for the procedure (34, 46, 50, 52) while the rest performed it on a 3T machine.

**Table 3:** Treatment parameters [^*^For descriptive purposes, the cohort of patients reported by Krishna et al. (35) and Elias et al. (47) were split into two groups, each with its own distinct characteristics].

**References**	**Mean skull density ratio ± SD (range)**	**No. of sonications ± SD (range)**	**Maximum energy delivered ± SD (range) (in Joules)**	**Peak temperature ± SD (range) (in °C)**	**Mean operative time ± SD (range) (in minutes)**	**MRI**
Lipsman et al. (24)	NA	22.5 ± 7.6 (12–29)	NA	59.3 ± 2.9 (56–63)	NA	3T
Elias et al. (25)	NA	17.9 ± 4.6 (11–26)	10,320 ± 4,537 (6,500–20,800)	58.5 ± 2.5 (54–63)	NA	3T
Chang et al. (26)	NA	NA	NA	53 ± 3.3 (48–61)	227.5 (169–293) (No vertigo group) to 260.6 (160–354) (Vertigo group)	3T
Gallay et al. (27)	NA	NA	16,073 ± 6,037	NA	285 ± 66	3T
Elias et al. (47) (Treatment group)^*^	NA	18.5 ± 5.2	14,497.0 ± 6,695.7 (3,500–34,860)	55.6 ± 2.3 (50.0–60.7)	NA	3T
Elias et al. (47) (Sham Crossover)^*^	NA	NA	NA	NA	NA	3T
Chang et al. (48)	NA	18.5 ± 5.2	14,497.0 ± 6,695.7 (3,500–34,860)	55.6 ± 2.3 (50.0–60.7)	NA	3T
Halpern et al. (49)	NA	18.5 ± 5.2	14,497.0 ± 6,695.7 (3,500–34,860)	55.6 ± 2.3 (50.0–60.7)	NA	3T
Zaroor et al. (28)	NA	20.8 ± 6.4	12,231.5 ± 3,189.8	56.88 ± 2.5	NA	3T
Schreglmann et al. (29)	NA	11 ± 3.2 (8–17)	12,008 ± 4,441 (7,800–19,950)	62.0 ± 2.5 (58–64)	271.6 ± 40 (215–305)	3T
Kim et al. (30)	NA	NA	NA	NA	NA	3T
Chazen et al. (31)	NA	NA	NA	NA	NA	3T
Federau et al. (32)	NA	18.6 ± 5.7 (12–28)	NA	NA	NA	3T
Jung et al. (33)	NA	16.8 (13–20)	15,910 ± 5,702.7	57.9	NA	3T
Iacopino et al. (34)	NA	NA	NA	NA	NA	1.5T
Krishna et al. (35)	0.54 ± 0.1	13.9 ± 4.5	NA	NA	174.3 ± 41.6 (Sonification time : 82.8 ± 30.8)	3T
Boutet et al. (36)	0.48 ± 0.1	NA	NA	56.6 ± 2.3	NA	3T
Park et al. (37)	0.49 ± 0.08 (0.26–0.6)	17.3 ± 1.6 (15–20)	15,552.4 ± 6,574.1 (7,150–25,488)	NA	NA	3T
Hori et al. (38)	Median 0.38 (0.27–0.61)	Median 17 (9–26)	Median 23,054 (5,849–38,658)	Median 56 (52–59)	NA	3T
Pineda-Pardo et al. (39)	NA	NA	NA	NA	NA	3T
Yang et al. (50)	NA	14	16,080	64	NA	1.5T
Jones et al. (40)	NA	NA	NA	NA	NA	3T
Sinai et al. (41)	Median 0.47 (0.31–0.67)	Median 19.5 (9–36)	Median 12,077 (6,000–35,500)	NA	NA	3T
Chang et al. (42)	0.51 ± 0.08 (0.26–0.72)	15.12 ± 3.88	NA	58.76 ± 2.89	NA	3T
Miller et al. (43)	NA	NA	NA	NA	NA	3T
Krishna et al. (44) (Pivotal)^*^	0.55 ± 0.1 (unreported for 17 pts)	17.4 ± 4.3	14,410 ± 7,390	55.6 ± 2.8	88 ± 40	3T
Krishna et al. (44) (Post Pivotal)^*^	0.5 ± 0.1 (unreported for 4 pts)	17.1 ± 5.3	16,910 ± 8,340	56.7 ± 2.5	105 ± 55	3T
Gallay et al. (45)	0.54 ± 0.06 (0.33–0.62)	NA	13,720 (5,850–36,000)	NA	NA	3T
Paff et al. (51)	0.65	13	18,302	59	80	3T
Buch et al. (52)	0.46	16	22,559	60	NA	1.5T
Fukutome et al. (46)	0.45 ± 0.11 (0.30–0.80)	NA	16,275 ± 8,610 (4,791–33,018)	57.3 ± 1.9 (54–60)	NA	1.5T

### Tremor Outcome

**Figure 2:**
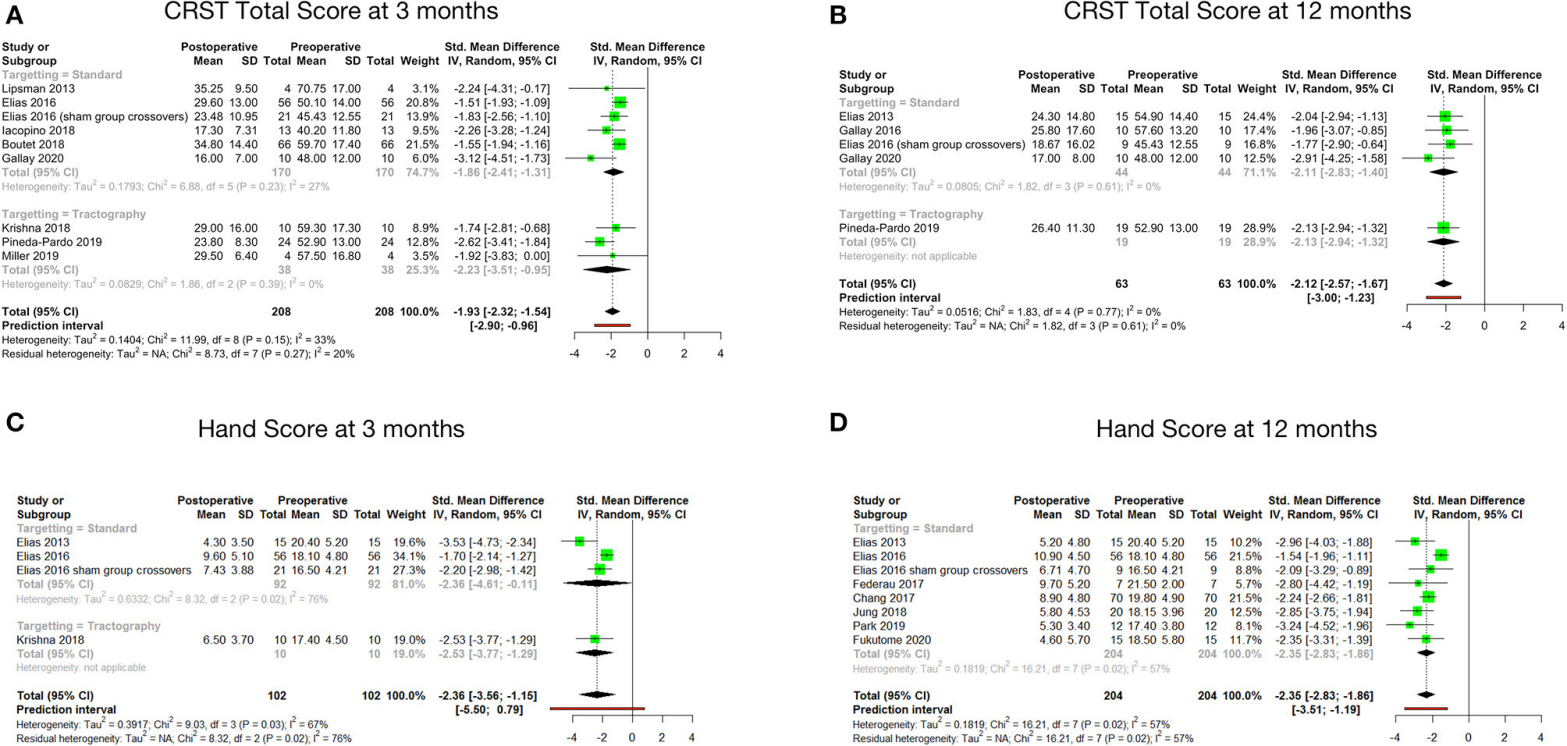
Pooled standard mean difference between preoperative and postoperative total CRST score at 3 months **(A)**, total CRST score at 12 months **(B)**, hand score at 3 months **(C)** and hand score at 12 months **(D)**.

Tremor outcomes, in the form of CRST scores and its subsets, for all studies have been summarized in Table 4. Total CRST scores 3 months after the procedure were reported by nine studies. The pooled standard mean difference between postoperative and preoperative total CRST score at 3 months was −1.93 (95% CI: −2.32 to −1.54, *p*-value < 0.001). The studies showed moderate heterogeneity with *I*^2^ of 33% (Figure 2A).

**Table 4:** Summary of outcomes after meta-analysis.

**Outcome variables**	**Standard mean difference between pre & postoperative score (95% CI)**	**No. of participants (studies)**	***p*-value**	**Heterogeneity (*I*^**2**^)**
Total CRST score (at 3 months)	−1.93 (−2.32 to −1.54)	208 (9)	<0.001^*^	Moderate (33%)
Total CRST score (at 12 months)	−2.12 (−2.57 to −1.67)	63 (5)	0.002^*^	Low (0%)
Hand score (at 3 months)	−2.36 (−3.56 to −1.15)	102 (4)	0.03^*^	Moderate (67%)
Hand score (at 12 months)	−2.35 (−2.83 to −1.86)	204 (8)	0.02^*^	Moderate (57%)
CRST Part C score (at 3 months)	−2.66 (−3.53 to −1.79)	104 (5)	0.08	Moderate (52%)
CRST Part C score (at 12 months)	−2.57 (−3.33 to −1.80)	202 (7)	0.01^*^	Moderate (64%)
QUEST score (at 3 months)	−1.49 (−2.93 to −0.04)	79 (3)	0.13	Moderate (51%)
QUEST score (at 6 months)	−2.20 (−3.40 to −1.00)	58 (4)	0.07	Moderate (57%)

*CI, confidence interval; NA, not applicable*.

* *Significant. Bold denote significant values*.

Seven studies reported total CRST scores at 12 months after the procedure. The pooled standard mean difference was −2.07 (95% CI: −2.70 to −1.44). *P*-value was found to be significant at <0.01. The studies showed high heterogeneity with *I*^2^ of 68%. Sensitivity analysis was done, and 2 studies (33, 47) were found to be contributing to heterogeneity. Analysis was redone after removing these 2 studies. Hence, the final analysis for total CRST scores at 12 months after the procedure included five studies. The pooled standard mean difference was −2.12 (95% CI: −2.57 to −1.67). *P*-value was found to be significant at 0.002. The studies showed low heterogeneity with *I*^2^ of 0% (Figure 2B).

Four cohorts reported hand scores (out of a total of 32) at 3 months. The pooled standard mean difference was −2.36 (95% CI: −3.56 to −1.15; *p*-value - 0.03). The studies showed high heterogeneity with *I*^2^ of 67% (Figure 2C). Eight cohorts reported hand scores at 12 months. The pooled standard mean difference was −2.35 (95% CI: −2.83 to −1.86; *p*-value - 0.02). The studies showed moderate heterogeneity with *I*^2^ of 57% (Figure 2D).

The standard mean difference between the preoperative and postoperative total CRST score and hand scores was found to be significant at 3 and 12 months following the procedure. Subgroup analysis of the mean changes in CRST scores according to the targeting technique (standard vs. DTI based) revealed that the difference was not statistically significant between the two groups.

### Disability and QOL Outcome

**Figure 3:**
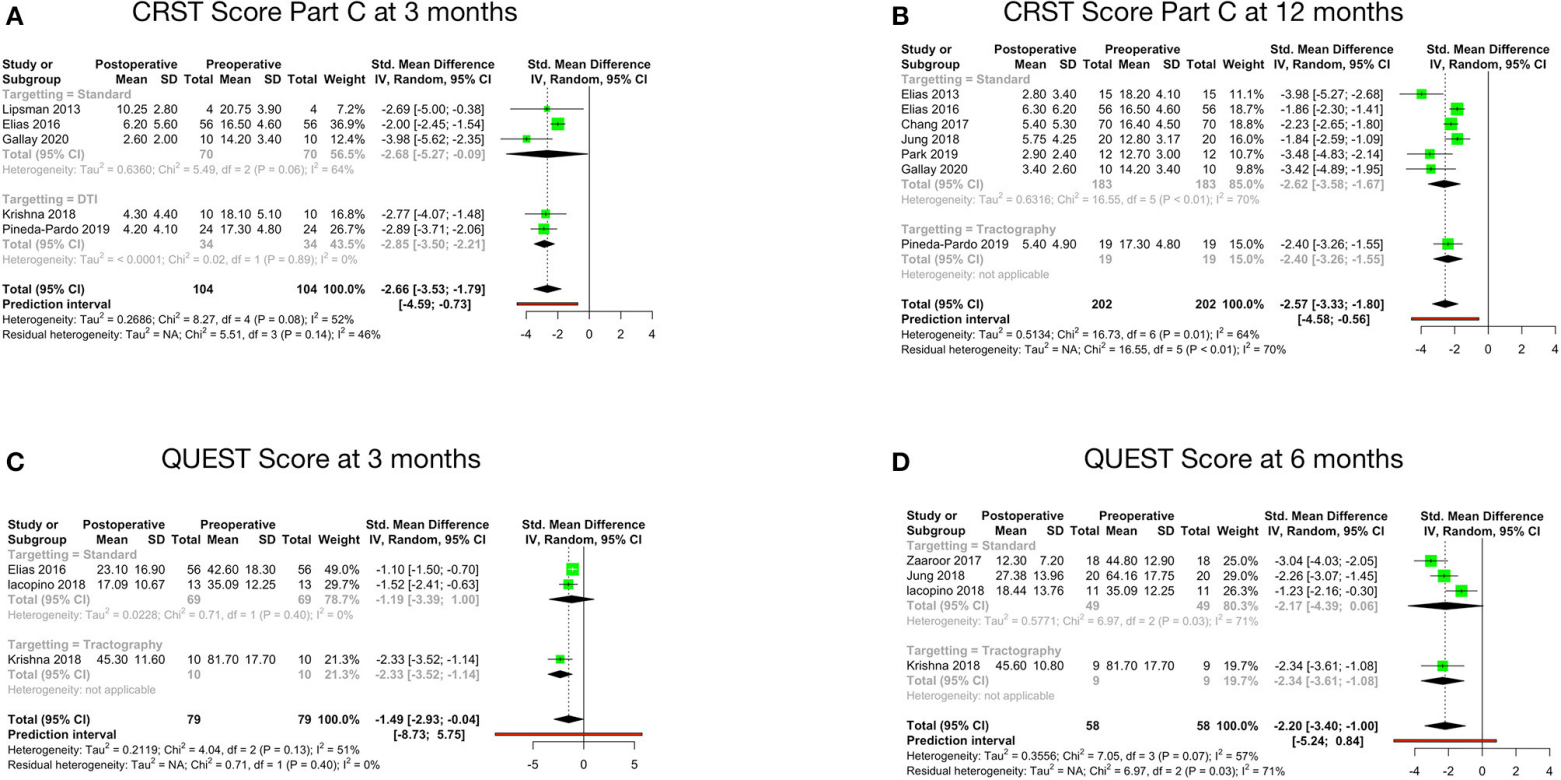
Pooled standard mean difference between preoperative and postoperative disability scores at 3 months **(A)**, disability scores at 12 months **(B)**, QUEST scores at 3 months **(C)** and QUEST scores at 6 months **(D)**.

Disability, as per the CRST Part C score at 3 months after MRgFUS, was reported by five studies. The pooled standard mean difference was −2.66 with 95% CI: −3.53 to −1.79 (*p*-value - 0.08). The studies showed moderate heterogeneity with *I*^2^ of 52% (Figure 3A). Disability at 12 months after MRgFUS was reported by eight cohorts. The pooled standard mean difference was −4.54 with 95% CI: −8.95 to −0.12 (*p*-value < 0.01). The studies showed considerable heterogeneity with *I*^2^ of 96%. Sensitivity analysis was done, and 1 study (42) was found to be contributing to heterogeneity. Analysis was redone after removing this study. Hence, the final analysis for disability at 12 months after MRgFUS included seven studies. The pooled standard mean difference was −2.57 with 95% CI: −3.33 to −1.80 (*p*-value - 0.01). The studies showed moderate heterogeneity with *I*^2^ of 64% (Figure 3B).

QOL, as per the QUEST score at 3 months, was reported by three cohorts. The pooled standard mean difference was −1.49 (95% CI: −2.93 to −0.04; *p*-value - 0.13). The studies showed moderate heterogeneity with *I*^2^ of 51% (Figure 3C). Four cohorts reported QOL at 6 months. The pooled standard mean difference was −2.20 (95% CI: −3.40 to −1.00; *p*-value - 0.07). The studies showed moderate heterogeneity with *I*^2^ of 57% (Figure 3D).

The comparison between preoperative and postoperative disability revealed statistically significant difference in the Part C score at 12 months (*p*-value: 0.01) (Table 4). Further subgroup analysis disclosed no statistically significant difference.

### Complications

**Figure 4:**
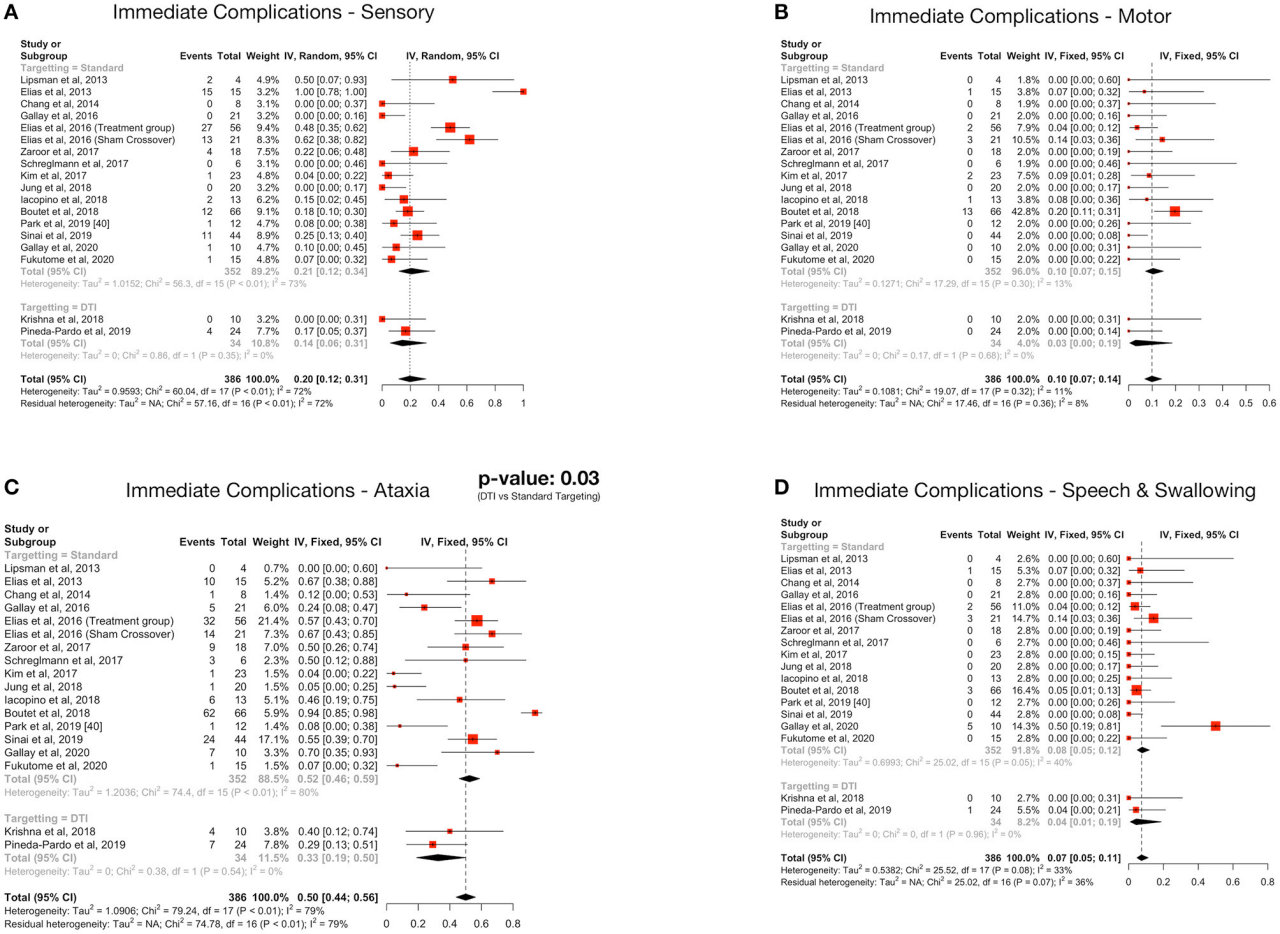
Forest plots depicting pooled proportions of immediate sensory **(A)**, motor **(B)**, ataxia **(C)** and speech and swallowing **(D)** related complications.

Details regarding the immediate, short term, and long-term complications are provided in the supplementary data (Supplementary Materials 2–4). The total complications were arranged according to the targeting method – standard vs. DTI based (Table 5) The pooled proportion of sensory, motor, ataxia and speech & swallowing related complications was calculated for immediate, early and late (occurring or persisting after 3 months) complications (Table 6, Figure 4, Supplementary Material 5).

**Table 5:** Total number of complications (grouped according to the targeting method).

**References**	**Neurological**												**Minor/Treatment Related**											
	**Sensory**			**Ataxia/gait disturbance**			**Motor**			**Speech and swallowing**			**Headache and fatigue**			**Sonication related**			**Frame and MRI related**			**Other**		
	**A**	**B**	**C**	**A**	**B**	**C**	**A**	**B**	**C**	**A**	**B**	**C**	**A**	**B**	**C**	**A**	**B**	**C**	**A**	**B**	**C**	**A**	**B**	**C**
**Atlas based targeting**																								
Lipsman et al. (24)	2	1	NA	0	0	NA	0	0	NA	0	0	NA	0	0	NA	0	0	NA	0	0	NA	1 Deep Vein Thrombosis	1 Deep Vein Thrombosis	NA
Elias et al. (25)	15	4	4	10	5	0	1 (Grip)	1 (5 days)	0	1	0	0	0	0	0	33	0	0	12	0	0	0	0	0
Chang et al. (26)	0	0	0	1	1	0	0	0	0	0	0	0	0	0	0	5	0	0	0	0	0	3 failed to attain temperature above 50 °C	0	0
Gallay et al. (27)	0	0	0	5	5	1	0	0	0	0	0	0	0	0	0	0	0	0	0	0	0	0	0	0
Elias et al. (47) (Treatment group)	27	17	10	32	17	8	2 (Grip)	2 (Grip)	1 (Grip)	2	2	2	11	3	2	54	0	0	17	0	0	0	0	0
Elias et al. (47) (Sham Crossover)	13	8	8	14	7	4	3 (Grip)	2 (Grip)	1 (Grip)	3	2	2	11	2	2	16	0	0	7	0	0	0	0	0
Chang et al. (48)	NA	NA	1	NA	NA	12	NA	NA	1	NA	NA	0	NA	NA	0	NA	NA	0	NA	NA	0	NA	NA	0
Halpern et al. (49)	NA	NA	12	NA	NA	10	NA	NA	2	NA	NA	1	NA	NA	1	NA	NA	0	NA	NA	0	NA	NA	1-slow movements
Zaroor et al. (28)^#^	4	4	0	9	9	0	0	0	0	0	0	0	4	0	0	39	0	0	8	8	0	0	0	0
Schreglmann et al. (29)	0	0	0	3	3	0	0	0	0	0	0	0	0	0	0	4	0	0	0	0	0	0	0	0
Kim et al. (30)	1	1	0	1	1	0	2 (Facial)	2 (Facial- one resolved in 1 month)	1 (Facial)	0	0	0	0	0	0	0	0	0	0	0	0	0	0	0
Federau et al. (32)	NA	NA	NA	NA	NA	NA	NA	NA	NA	NA	NA	NA	NA	NA	NA	NA	NA	NA	NA	NA	NA	NA	NA	NA
Jung et al. (33)	0	0	0	1	1	0	0	0	0	0	0	0	0	0	0	10	0	0	3	0	0	0	0	0
Iacopino et al. (34)^#^	2	0	0	6	3	2	1 (Grip)	1 (Grip - 1 week)	0	0	0	0	0	0	0	10	0	0	6	0	0	4 ET patients - aborted treatment due to severe headache; 1 ET patient - failed to attain ablative temperature	0	0
Boutet et al. (36)	12	5	NA	62	20	NA	13	6	NA	3	3	NA	0	0	NA	NA	NA	NA	NA	NA	NA	0	0	NA
Park et al. (37)	1	1	0	1	1	1	0	0	0	0	0	0	0	0	0	2	0	0	0	0	0	0	0	0
Hori et al. (38)	NA	NA	NA	NA	NA	NA	NA	NA	NA	NA	NA	NA	NA	NA	NA	NA	NA	NA	NA	NA	NA	NA	NA	NA
Jones et al. (40)	NA	NA	NA	NA	NA	NA	NA	NA	NA	NA	NA	NA	NA	NA	NA	NA	NA	NA	NA	NA	NA	NA	NA	NA
Sinai et al. (41)	11	11	5	24	24	2	0	0	0	0	0	0	4	4	0	65	0	0	1	1	0	0	0	0
Chang et al. (42)	NA	NA	NA	NA	NA	NA	NA	NA	NA	NA	NA	NA	NA	NA	NA	NA	NA	NA	NA	NA	NA	NA	NA	NA
Krishna et al. (44) (Pivotal)	NA	NA	42	NA	NA	59	NA	NA	4 mild, 2 moderate	NA	NA	5	NA	NA	0	NA	NA	0	NA	NA	0	NA	NA	6 mild, 3 moderate
Krishna et al. (44) (Post Pivotal)	NA	NA	56	NA	NA	89	NA	NA	16 mild	NA	NA	17	NA	NA	0	NA	NA	0	NA	NA	0	NA	NA	16 mild, 2 moderate
Gallay et al. (45)	1	1	1	7	5	5	0	0	0	5	1	1	0	0	0	4	0	0	1	1	0	0	0	0
Paff et al. (51)	0	0	0	1	1	0	1 (c/l lower limb)	1 (c/l lower limb) (1 m)	0	0	0	0	0	0	0	0	0	0	0	0	0	0	0	0
Fukutome et al. (46)	1	1	1	1	1	1	0	0	0	0	0	0	0	0	0	9	0	0	0	0	0	0	0	0
**Total**	**90**	**54**	**140**	**178**	**104**	**194**	**23**	**15**	**28**	**14**	**8**	**28**	**30**	**9**	**5**	**251**	**0**	**0**	**55**	**10**	**0**	**9**	**1**	**28**
**DTI based targeting**																								
Chazen et al. (31)	NA	NA	NA	NA	NA	NA	NA	NA	NA	NA	NA	NA	NA	NA	NA	NA	NA	NA	NA	NA	NA	NA	NA	NA
Krishna et al. (35)	0	1	0	4	3	1	0	0	0	0	0	0	0	0	0	0	0	0	0	0	0	0	0	0
Pineda-Pardo et al. (39)	4	4	4	7	7	1	0	0	0	1	1	1	0	0	0	0	0	0	0	0	0	0	0	0
Yang et al. (50)	0	0	0	0	0	0	0	0	0	0	0	0	0	0	0	0	0	0	0	0	0	0	0	0
Miller et al. (43)	NA	NA	NA	NA	NA	NA	NA	NA	NA	NA	NA	NA	NA	NA	NA	NA	NA	NA	NA	NA	NA	NA	NA	NA
Buch et al. (52)	NA	NA	NA	NA	NA	NA	NA	NA	NA	NA	NA	NA	NA	NA	NA	NA	NA	NA	NA	NA	NA	NA	NA	NA
**Total**	**4**	**5**	**4**	**11**	**10**	**2**	**0**	**0**	**0**	**1**	**1**	**1**	**0**	**0**	**0**	**0**	**0**	**0**	**0**	**0**	**0**	**0**	**0**	**0**

*Studies reporting zero complications are marked as “0.” Studies in which no complication data was reported for respective time period are marked as “NA.”*

*A, immediate (during treatment to within 48 h); B, short term (48 h−3 months); C, long term (persisting for more than 3 months). Period in brackets denotes time until when the complication persisted*.

# *Complications not mentioned separately for ET patients*.

**Table 6:** Summary of complications after meta-analysis.

**Outcome variables**	**Pooled proportion (95% CI)**	**No. of participants (studies)**	**Heterogeneity (*I*^**2**^)**	***p*-value after subgroup analysis (standard vs. DTI based targeting)**
**Immediate**				
Sensory	20% (12–31%)	386 (18)	High (72%)	0.46
Motor	10% (7–14%)	386 (18)	Low (11%)	0.20
Ataxia	50% (44–56%)	386 (18)	High (79%)	0.03^*^
Speech & Swallowing	7% (5–11%)	386 (18)	Moderate (33%)	0.47
**Short-term**				
Sensory	16% (11–23%)	386 (18)	Moderate (43%)	0.93
Motor	6% (4–9%)	386 (18)	Low (0%)	0.46
Ataxia	29% (22–38%)	386 (18)	Moderate (49%)	0.95
Speech and swallowing	4% (3–7%)	386 (18)	Low (0%)	0.96
**Long-term**				
Sensory	13% (7–23%)	368 (16)	High (76%)	0.88
Motor	5% (3–7%)	391 (17)	Low (0%)	0.86
Ataxia	31% (24–38%)	378 (16)	High (87%)	0.09
Speech and swallowing	5% (3–8%)	391 (17)	Low (0%)	0.77

*CI, confidence interval*.

* *Significant. Bold denote significant values*.

Ataxia was the most common postoperative complication. All complications showed a decreasing trend over time. Subgroup analysis revealed significantly less immediate post procedure ataxia related complications in the DTI group, although no significant difference was noted over long term analysis.

## Discussion

The first reports of the use of MRgFUS in medically refractory ET were published in 2013 (24, 25). Standard atlas-based targeting was utilized to create a lesion in the contralateral VIM nucleus of the thalamus. The USFDA approved the use of MRgFUS in ET in 2016 after a randomized sham-controlled trial showed favorable results in the MRgFUS group (47). In a short time period, there has been a significant amount of research on the subject. However, most of these studies have small sample size. Our review article summarizes the latest available evidence in literature in terms of efficacy and complications of MRgFUS for ET. Owing to the paucity of studies involving large number of patients, this meta-analysis strives to provide pooled results of a number of smaller studies on the topic. We did an updated systematic review and meta-analysis of the studies describing the outcomes and adverse events following the use of MRgFUS in essential tremor. Though reviews have been published in the past on this topic, but there were significant shortcomings (15, 16). Our review article summarizes the latest available evidence in literature in terms of efficacy and complications of MRgFUS for ET. The primary outcome analyzed was the change in total CRST score and hand score (out of 32) after treatment, while the secondary outcomes measured were the quality of life and the complication rates. We have also tried to find whether any difference in efficacy and complication rate exists according to the area targeted - VIM nucleus of thalamus or CTT in the PSA.

### Clinical Efficacy

All studies have reported good postoperative outcomes. We found a significant difference in the pooled SMD between the postoperative and preoperative primary outcome variables, at 3- and 12-months post-procedure. This shows MRgFUS to be an effective surgical modality for the treatment of ET. Additionally, there was a statistically significant improvement in the disability of the patients at 12 months postoperatively, as evaluated by the CRST Part C score. Only one study had a control group; hence between-group comparison was not possible.

For any other surgical technique to replace DBS as the procedure of choice for refractory ET, it has to prove itself as at-par, if not better than DBS. Comparative studies between RFA and DBS have reported better improvement in function and fewer adverse effects with DBS (53). Gamma knife thalamotomy for ET was first described in the 1990s. It's a non-invasive procedure, however, the inability to monitor real-time clinical response, variation in the size of the lesion produced, unpredictable radiation effects, and a delay in clinical response have resulted in GKT being reserved for patients who are otherwise unfit for DBS (12).

Non-invasiveness of the MRgFUS is an advantage of MRgFUS over DBS. Class I evidence in the form of an RCT gave a big impetus to MRgFUS (47). In a retrospective analysis of RFA, DBS, and MRgFUS for ET, outcomes of the procedures between the three groups were not statistically different (30). Another retrospective analysis showed comparable efficacy and QOL between unilateral DBS and MRgFUS (54). A recent study compared a trial on the use of VIM DBS for ET, with the RCT done by Elias et al. (47, 55, 56). They found a greater percentage improvement with DBS, although the patients in the DBS group had worse baseline tremor scores.

### Long Term Outcome

Sustained improvement in tremor scores has been demonstrated on long term follow up of patients (37, 41, 48, 49). At 3 years of follow up, the patients enrolled in the RCT had a reduction of 56% in hand score, 63% in disability score and a 50% improvement in the QOL (49). At 4 years of follow up in 12 patients, a 56% reduction in hand score and 63% reduction in the disability scores was seen (37). The maximum available follow up of 5 years in two patients revealed a total CRST score of 8.0 (6–10) and QUEST score of 11.0 (6–16), as compared to a baseline score of 46.0 (16–74) and 41.5 (15–93) respectively (41).

A decline in efficacy over time, in the form of a small increase in the hand tremor and disability scores at 3 years as compared to the scores at 6 months has been noted (49). Four patients out of 76 underwent DBS (49). Sinai et al. observed a return of tremor in 11% of their patients (5/44) (41). Further studies with a greater number of patients are needed to refute this observation. The decline in the efficacy over time may be due to the progressive nature of the disease (57). In such cases, it is feasible to treat the patients again and this is certainly a big strength of MRgFUS.

### Treatment Parameters

A meta-analysis of the mentioned treatment parameters could not be done due to the unavailability of adequate data for analysis. Some studies in literature have tried to correlate various treatment parameters with the clinical outcome. SDR was significantly associated with the outcome at 1 and 6 months by Sinai et al. (41), while no such relationship was found in other studies (42, 44, 46). The study which included patients with SDR < 0.4 found no statistically significant difference in the mean SDR of patients who had sustained improvement in symptoms and those who had recurrence of symptoms in this study. Traditionally, a SDR value of <0.40 has been associated with higher energy requirements. But recent clinical series investigating this topic have found no significant difference in the clinical outcome or the complication rate in this subgroup as compared to the patients with SDR value > 0.40 (58, 59).

The higher maximal temperature has been found to significantly influence the percentage change in tremor scores (41, 45). Intraoperative tremor reduction has not been found to correlate to outcome at 3 months, while procedure duration and number of sonications have been shown to be significantly less with the use of DTI (35). Studies have previously found an association between younger age, short disease duration, better baseline tremor scores, fewer number of sonications and a higher maximal temperature during treatment to a better outcome (35, 44, 46). Experience acquired with the technique has also been found to have a positive impact on the outcome positively (44). It has been recommended by some that the procedure should be restricted to a few specialized centers only (41). Barring three patients who underwent bilateral procedures 1 year apart (27), all FUS procedures have been performed unilaterally. ET is a progressive disease, with most patients having bilateral symptoms (57). Thus, more experience with bilateral procedures is required.

### Complications

More than 1/3rd of patients developed sonication related complications, amongst which head pain and dizziness were the most common. This seems to be a significant source of discomfort for the patient. Iacopino et al. (34) reported four patients in whom treatment had to be aborted due to severe head pain. None of the sonication or frame related complications persisted beyond 3 months.

Ataxia, which included gait disturbance and hand ataxia, was the most common neurological side effect, followed by sensory deficits. The immediate pooled proportion of ataxia was 50%, while it was 20% for sensory complications, which is considerable. The high complication rate has been postulated to be due to the small size of the VIM nucleus and non-visibility on MRI resulting in the potential overlap of the lesion with the surrounding structures like the ML and CST. The reason why ataxia is the most common acute complication was suggested to be due to the objective nature of assessment (36). Further, it was demonstrated that the area responsible for postoperative ataxia overlapped significantly with the area associated with clinical benefit (36). Fortunately, data suggests that these complications partially resolve with time as evident by the decreased incidence of late complications (Table 4). Furthermore, no additional side effects were observed in each subsequent year of follow up amongst the patients enrolled in the RCT (37, 48, 49). But a major limitation in assessing the long-term complications of the patients undergoing MRgFUS is the high dropout rate (48, 49). Halpern et al. found it to be as high as 31% in 3 years (49). Thus, a true picture of the permanence of the complications is hard to assess.

No hemorrhage, seizure or trajectory related complications have been noted till now with MRgFUS for ET, making it a uniquely safe procedure in this aspect as compared to DBS or RFA (11, 30, 59).

### Role of DTI in Target Localization Technique

VIM nucleus of the thalamus, which has traditionally been used as the target for MRgFUS, is not visible on the 3T MRI and surgeons have to target it based on an atlas or an estimate based on their experience. Microelectrode recording is not possible intra-operatively as the procedure is incisionless. A series of low power sonications, delivered before creating the actual lesion, are usually used to observe the resolution of tremor or the appearance of side effects. However, this method of confirming target accuracy has not been scientifically validated. On the other hand, CTT fibers localized to the PSA have been shown to have a high density of clinically relevant fibers for targeting (53, 60, 61). Gallay et al. were the first to target the CTT using atlas-based co-ordinates (27). Boutet et al. identified distinct areas in the thalamus associated with clinical benefit and complications (36).

Advances in DTI have allowed surgeons to visualize the CTT and individualize surgical targeting accordingly. Both the CST and ML can also be localized, thus clearly demarcating the target according to the unique anatomy of each patient (31, 35, 39, 43). The surgical target thus identified has been found to be anterior and lateral to the atlas-based target (62). This should theoretically lead to better postoperative outcomes. Our analysis revealed a significant reduction in ataxia immediately after DTI based targeting. This is noteworthy as post procedure ataxia has been observed to be an important source of patient discomfort. Thus, use of DTI could decrease patient distress and lead to better acceptability of the procedure. A significant benefit was not apparent on analysis of the other complications. This could be due to the smaller number of studies utilizing DTI. Moreover, it must be emphasized that DTI based targeting is not yet standardized. Differences exist in the number of tracts generated for localization. All the centers have generated the ipsilateral CTT tract (one track) for target localization in the VIM region of the thalamus. Many studies have additionally utilized the ipsilateral CST and ML tracts (three tracks) and adjusted the surgical target accordingly to avoid significant motor and sensory side effects. Anatomical considerations of the CTT tract which originates from the contralateral dentate projections and then decussates in the superior cerebellar peduncle to reach the ipsilateral motor cortex via the thalamus, have led some centers to generate the contralateral CTT tract (four track) in addition to the previously defined three tracks, for refining the target localization (62, 63). Thus, there are differences between the various surgical teams performing DTI based MRgFUS and there is no consensus on what are the best practices although there seems to be an increasing trend to the DTI usage (62–65). The utility of DTI in this regard remains to be definitely proven with additional numbers and long-term data.

### Limitations

Observational studies formed the majority basis for the analysis as there is only one clinical trial on the subject. These two types of studies are assessed differently in terms of bias and the strength of evidence, and thus recommendations, that they can offer are markedly different. Authors have used various subsets of the CRST scale to report the outcomes, thus precluding standardized comparison. Few studies included patients who underwent bilateral MRgFUS, which is associated with higher rates of complication. However, the data of these patients was not provided separately and could not be excluded from our analysis. The possibility of some overlap between subsets of patients reported from the same center cannot be completely ruled out. DTI based targeting is a novel procedure, and the number of studies utilizing it are quite low in number. A high level of heterogeneity in between studies needs to be kept in mind.

## Conclusion

MRgFUS for ET is an effective procedure for relieving unilateral tremor. Use of DTI based targeting revealed a significant reduction in post procedure ataxia related complications as compared to traditional targeting techniques. Analysis of other complications further revealed a decreasing trend on follow up. As of now, it seems to be the procedure of choice for patients unable to tolerate an invasive procedure. For it to replace established surgical options like DBS, further research will be required to prove long term clinical efficacy in both unilateral and bilateral procedures.

## Data Availability Statement

The original contributions presented in the study are included in the article/Supplementary Material, further inquiries can be directed to the corresponding author/s.

## Author Contributions

MA and KG: conceptualization, data curation, formal analysis, investigation, methodology, resources, software, roles/writing - original draft, and writing - review and editing. RS, RR, and VN: data curation, visualization, and writing - review and editing. MS: project administration, supervision, and writing - review and editing. All authors contributed to the article and approved the submitted version.

## Conflict of Interest

The authors declare that the research was conducted in the absence of any commercial or financial relationships that could be construed as a potential conflict of interest.

## Abbreviations

MRgFUS: magnetic resonance guided focused ultrasound
ET: essential tremor
RFA: radiofrequency ablation
VIM: ventral intermediate
DBS: deep brain stimulation
GKT: gamma knife thalamotomy
MRI: magnetic resonance imaging
DTI: diffusion tensor imaging
CTT: cerebello thalamic tract
PRISMA: Preferred Reporting Items for Systematic Reviews and Meta Analyses
CRST: clinical rating scale for tremor
QOL: quality of life
QUEST: quality of life in essential tremor
SDR: skull density ratio
RCT: randomized controlled trial
PSA: posterior subthalamic area
CST: corticospinal tract
ML: medial lemniscus
SMD: standardized mean difference
SDC: supplementary digital content.

## References

[1] Deuschl G Bain P Brin M. Consensus statement of the movement disorder society on tremor. Ad Hoc Scientific Committee. Mov Disord. (1998) 13 (Suppl. 3):2–23. 10.1002/mds.870131303 9827589

[2] Bhatia KP Bain P Bajaj N Elble RJ Hallett M Louis ED. Consensus statement on the classification of tremors. from the task force on tremor of the International Parkinson and Movement Disorder Society. Mov Disord. (2018) 33:75–87. 10.1002/mds.27121 29193359 PMC6530552

[3] Louis ED Ferreira JJ. How common is the most common adult movement disorder? Update on the worldwide prevalence of essential tremor. Mov Disord. (2010) 25:534–41. 10.1002/mds.22838 20175185

[4] Kamble N Pal PK. Tremor syndromes: a review. Neurol India. (2018) 66 (Suppl. S1):36–47 10.4103/0028-3886.226440 29503326

[5] Deuschl G Raethjen J Hellriegel H Elble R. Treatment of patients with essential tremor. Lancet Neurol. (2011) 10:148–61. 10.1016/S1474-4422(10)70322-7 21256454

[6] Schreglmann SR Krauss JK Chang JW Martin E Werner B Bauer R. Functional lesional neurosurgery for tremor: back to the future? J Neurol Neurosurg Psychiatry. (2018) 89:727–35. 10.1136/jnnp-2017-316301 29269505

[7] Benabid AL Pollak P Gervason C Hoffmann D Gao DM Hommel M. Long-term suppression of tremor by chronic stimulation of the ventral intermediate thalamic nucleus. Lancet. (1991) 337:403–6. 10.1016/0140-6736(91)91175-T 1671433

[8] Benabid AL Pollak P Gao D Hoffmann D Limousin P Gay E. Chronic electrical stimulation of the ventralis intermedius nucleus of the thalamus as a treatment of movement disorders. J Neurosurg. (1996) 84:203–14. 10.3171/jns.1996.84.2.0203 8592222

[9] Zesiewicz TA Elble RJ Louis ED Gronseth GS Ondo WG Dewey RB Jr. Evidence-based guideline update: treatment of essential tremor: report of the quality standards subcommittee of the American Academy of Neurology. Neurology. (2011) 77:1752–5. 10.1212/WNL.0b013e318236f0fd 22013182 PMC3208950

[10] Sankhe M Churi O. Newer advances in lesional surgery for movement disorders. Neurol India. (2018) 66 (Suppl. S1):113–21 10.4103/0028-3886.226463 29503333

[11] Wong JK Hess CW Almeida L Middlebrooks EH Christou EA Patrick EE. Deep brain stimulation in essential tremor: targets, technology, and a comprehensive review of clinical outcomes. Expert Rev Neurother. (2020) 20:319–31. 10.1080/14737175.2020.1737017 32116065 PMC7174089

[12] Schreglmann SR Krauss JK Chang JW Bhatia KP Kägi G. Functional lesional neurosurgery for tremor: a systematic review and meta-analysis. J Neurol Neurosurg Psychiatry. (2018) 89:717–26. 10.1136/jnnp-2017-316302 29326290

[13] Harary M Segar DJ Huang KT Tafel IJ Valdes PA Cosgrove GR. Focused ultrasound in neurosurgery: a historical perspective. Neurosurg Focus. (2018) 44:E2. 10.3171/2017.11.FOCUS17586 29385919

[14] Ranjan M Elias GJB Boutet A Zhong J Chu P Germann J. Tractography-based targeting of the ventral intermediate nucleus: accuracy and clinical utility in MRgFUS thalamotomy. J Neurosurg. (2019) 1–8. 10.3171/2019.6.JNS19612. [Epub ahead of print]. 31561221

[15] Mohammed N Patra D Nanda A. A meta-analysis of outcomes and complications of magnetic resonance-guided focused ultrasound in the treatment of essential tremor. Neurosurg Focus. (2018) 44:E4. 10.3171/2017.11.FOCUS17628 29385917

[16] Schreglmann SR Bhatia KP Hägele-Link S Werner B Martin E Kägi G. Letter to the Editor. Errors in the meta-analysis of outcomes and complications of MRgFUS. Neurosurg Focus. (2018) 45:E15. 10.3171/2018.3.FOCUS1898 29961390

[17] Fahn S Tolosa E Marin C. Clinical rating scale for tremor. In: Jankovic J Tolosa E editors. Parkinson's Disease and Movement Disorders. Baltimore, MD: Urban and Schwarzenberg (1988). p. 225–34.

[18] Stacy MA Elble RJ Ondo WG Wu SC Hulihan J. Assessment of interrater and intrarater reliability of the Fahn-Tolosa-Marin tremor rating scale in essential tremor. Mov Disord. (2007) 22:833–8. 10.1002/mds.21412 17343274

[19] Viechtbauer W. Conducting meta-analyses in R with the metafor package. J Stat Softw. (2010) 36:1–48. 10.18637/jss.v036.i03

[20] Balduzzi S Rücker G Schwarzer G. How to perform a meta-analysis with R: a practical tutorial. Evid Based Ment Health. (2019) 22:153–60. 10.1136/ebmental-2019-300117 31563865 PMC10231495

[21] Wickham H. ggplot2: Elegant Graphics for Data Analysis. New York, NY: Springer-Verlag (2016).

[22] Higgins JP Thompson SG Deeks JJ Altman DG. Measuring inconsistency in meta-analyses. BMJ. (2003) 327:557–60. 10.1136/bmj.327.7414.557 12958120 PMC192859

[23] Slim K Nini E Forestier D Kwiatkowski F Panis Y Chipponi J. Methodological index for non-randomized studies (minors): development and validation of a new instrument. ANZ J Surg. (2003) 73:712–6. 10.1046/j.1445-2197.2003.02748.x 12956787

[24] Lipsman N Schwartz ML Huang Y Lee L Sankar T Chapman M. MR-guided focused ultrasound thalamotomy for essential tremor: a proof-of-concept study. Lancet Neurol. (2013) 12:462–8. 10.1016/S1474-4422(13)70048-6 23523144

[25] Elias WJ Huss D Voss T Loomba J Khaled M Zadicario E. A pilot study of focused ultrasound thalamotomy for essential tremor. N Engl J Med. (2013) 369:640–8. 10.1056/NEJMoa1300962 23944301

[26] Chang WS Jung HH Kweon EJ Zadicario E Rachmilevitch I Chang JW. Unilateral magnetic resonance guided focused ultrasound thalamotomy for essential tremor: practices and clinicoradiological outcomes. J Neurol Neurosurg Psychiatry. (2015) 86:257–64. 10.1136/jnnp-2014-307642 24876191

[27] Gallay MN Moser D Rossi F Pourtehrani P Magara AE Kowalski M. Incisionless transcranial MR-guided focused ultrasound in essential tremor: cerebellothalamic tractotomy. J Ther Ultrasound. (2016) 4:5. 10.1186/s40349-016-0049-8 26877873 PMC4752806

[28] Zaaroor M Sinai A Goldsher D Eran A Nassar M Schlesinger I. Magnetic resonance-guided focused ultrasound thalamotomy for tremor: a report of 30 Parkinson's disease and essential tremor cases. J Neurosurg. (2018) 128:202–10. 10.3171/2016.10.JNS16758 28298022

[29] Schreglmann SR Bauer R Hägele-Link S Bhatia KP Natchev P Wegener N. Unilateral cerebellothalamic tract ablation in essential tremor by MRI-guided focused ultrasound. Neurology. (2017) 88:1329–33. 10.1212/WNL.0000000000003795 28275083

[30] Kim M Jung NY Park CK Chang WS Jung HH Chang JW. Comparative evaluation of magnetic resonance-guided focused ultrasound surgery for essential tremor. Stereotact Funct Neurosurg. (2017) 95:279–86. 10.1159/000478866 28810261

[31] Chazen JL Sarva H Stieg PE Min RJ Ballon DJ Pryor KO. Clinical improvement associated with targeted interruption of the cerebellothalamic tract following MR-guided focused ultrasound for essential tremor. J Neurosurg. (2018) 129:315–23. 10.3171/2017.4.JNS162803 29053074

[32] Federau C Goubran M Rosenberg J Henderson J Halpern CH Santini V. Transcranial MRI-guided high-intensity focused ultrasound for treatment of essential tremor: a pilot study on the correlation between lesion size, lesion location, thermal dose, and clinical outcome. J Magn Reson Imaging. (2018) 48:58–65. 10.1002/jmri.25878 29076274

[33] Jung NY Park CK Chang WS Jung HH Chang JW. Effects on cognition and quality of life with unilateral magnetic resonance-guided focused ultrasound thalamotomy for essential tremor. Neurosurg Focus. (2018) 44:E8. 10.3171/2017.11.FOCUS17625 29385928

[34] Iacopino DG Gagliardo C Giugno A Giammalva GR Napoli A Maugeri R. Preliminary experience with a transcranial magnetic resonance-guided focused ultrasound surgery system integrated with a 1.5-T MRI unit in a series of patients with essential tremor and Parkinson's disease. Neurosurg Focus. (2018) 44:E7. 10.3171/2017.11.FOCUS17614 29385927

[35] Krishna V Sammartino F Agrawal P Changizi BK Bourekas E Knopp MV. Prospective tractography-based targeting for improved safety of focused ultrasound thalamotomy. Neurosurgery. (2019) 84:160–8. 10.1093/neuros/nyy020 29579287

[36] Boutet A Ranjan M Zhong J Germann J Xu D Schwartz ML. Focused ultrasound thalamotomy location determines clinical benefits in patients with essential tremor. Brain. (2018) 141:3405–14. 10.1093/brain/awy278 30452554

[37] Park YS Jung NY Na YC Chang JW. Four-year follow up results of magnetic resonance-guided focused ultrasound thalamotomy for essential tremor. Mov Disord. (2019) 34:727–34. 10.1002/mds.27637 30759322

[38] Hori H Yamaguchi T Konishi Y Taira T Muragaki Y. Correlation between fractional anisotropy changes in the targeted ventral intermediate nucleus and clinical outcome after transcranial MR-guided focused ultrasound thalamotomy for essential tremor: results of a pilot study. J Neurosurg. (2019) 132:568–73. 10.3171/2018.10.JNS18993 30771772

[39] Pineda-Pardo JA Martínez-Fernández R Rodríguez-Rojas R Del-Alamo M Hernández F Foffani G. Microstructural changes of the dentato-rubro-thalamic tract after transcranial MR guided focused ultrasound ablation of the posteroventral VIM in essential tremor. Hum Brain Mapp. (2019) 40:2933–42. 10.1002/hbm.24569 30865338 PMC6865586

[40] Jones RM Kamps S Huang Y Scantlebury N Lipsman N Schwartz ML. Accumulated thermal dose in MRI-guided focused ultrasound for essential tremor: repeated sonications with low focal temperatures. J Neurosurg. (2019) 132:1802–9. 10.3171/2019.2.JNS182995 31075781 PMC7139920

[41] Sinai A Nassar M Eran A Constantinescu M Zaaroor M Sprecher E. Magnetic resonance-guided focused ultrasound thalamotomy for essential tremor: a 5-year single-center experience. J Neurosurg. (2019) 1–8. 10.3171/2019.3.JNS19466. [Epub ahead of print]. 31277064

[42] Chang KW Park YS Chang JW. Skull factors affecting outcomes of magnetic resonance-guided focused ultrasound for patients with essential tremor. Yonsei Med J. (2019) 60:768–73. 10.3349/ymj.2019.60.8.768 31347332 PMC6660436

[43] Miller TR Zhuo J Eisenberg HM Fishman PS Melhem ER Gullapalli R. Targeting of the dentato-rubro-thalamic tract for MR-guided focused ultrasound treatment of essential tremor. Neuroradiol J. (2019) 32:401–7. 10.1177/1971400919870180 31407957 PMC6856993

[44] Krishna V Sammartino F Cosgrove R Ghanouni P Schwartz M Gwinn R. Predictors of outcomes after focused ultrasound thalamotomy. Neurosurgery. (2020) 87:229–37. 10.1093/neuros/nyz417 31690945

[45] Gallay MN Moser D Jeanmonod D. MR-guided focused ultrasound cerebellothalamic tractotomy for chronic therapy-resistant essential tremor: anatomical target reappraisal and clinical results. J Neurosurg. (2020) 1–10. 10.3171/2019.12.JNS192219. [Epub ahead of print]. 32032945

[46] Fukutome K Kuga Y Ohnishi H Hirabayashi H Nakase H. What factors impact the clinical outcome of magnetic resonance imaging-guided focused ultrasound thalamotomy for essential tremor? J Neurosurg. (2020) 1–6. 10.3171/2020.2.JNS192814. [Epub ahead of print]. 32357330

[47] Elias WJ Lipsman N Ondo WG Ghanouni P Kim YG Lee W. A randomized trial of focused ultrasound thalamotomy for essential tremor. N Engl J Med. (2016) 375:730–9. 10.1056/NEJMoa1600159 27557301

[48] Chang JW Park CK Lipsman N Schwartz ML Ghanouni P Henderson JM. A prospective trial of magnetic resonance-guided focused ultrasound thalamotomy for essential tremor: results at the 2-year follow-up. Ann Neurol. (2018) 83:107–14. 10.1002/ana.25126 29265546

[49] Halpern CH Santini V Lipsman N Lozano AM Schwartz ML Shah BB. Three-year follow-up of prospective trial of focused ultrasound thalamotomy for essential tremor. Neurology. (2019) 93:e2284–93. 10.1212/WNL.0000000000008561 31748250

[50] Yang AI Chaibainou H Wang S Hitti FL McShane BJ Tilden D. Focused ultrasound thalamotomy for essential tremor in the setting of a ventricular shunt: technical report. Oper Neurosurg. (2019) 17:376–81. 10.1093/ons/opz013 30888021

[51] Paff M Boutet A Neudorfer C Elias GJB Germann J Loh A. Magnetic resonance-guided focused ultrasound thalamotomy to treat essential tremor in nonagenarians. Stereotact Funct Neurosurg. (2020) 98:182–6. 10.1159/000506817 32224617 PMC7384340

[52] Buch VP McShane BJ Beatson N Yang A Blanke A Tilden D. Focused ultrasound thalamotomy with dentato-rubro-thalamic tractography in patients with spinal cord stimulators and cardiac pacemakers. Stereotact Funct Neurosurg. (2020) 98:263–9. 10.1159/000507031 32403106

[53] Schuurman PR Bosch DA Bossuyt PM Bonsel GJ van Someren EJ de Bie RM. A comparison of continuous thalamic stimulation and thalamotomy for suppression of severe tremor. N Engl J Med. (2000) 342:461–8. 10.1056/NEJM200002173420703 10675426

[54] Huss DS Dallapiazza RF Shah BB Harrison MB Diamond J Elias WJ. Functional assessment and quality of life in essential tremor with bilateral or unilateral DBS and focused ultrasound thalamotomy. Mov Disord. (2015) 30:1937–43. 10.1002/mds.26455 26769606

[55] Harary M Segar DJ Hayes MT Cosgrove GR. Unilateral thalamic deep brain stimulation versus focused ultrasound thalamotomy for essential tremor. World Neurosurg. (2019) 126:e144–52. 10.1016/j.wneu.2019.01.281 30794976

[56] Wharen RE Jr Okun MS Guthrie BL Uitti RJ Larson P Foote K. Thalamic DBS with a constant-current device in essential tremor: a controlled clinical trial. Parkinsonism Relat Disord. (2017) 40:18–26. 10.1016/j.parkreldis.2017.03.017 28400200

[57] Louis ED Agnew A Gillman A Gerbin M Viner AS. Estimating annual rate of decline: prospective, longitudinal data on arm tremor severity in two groups of essential tremor cases. J Neurol Neurosurg Psychiatry. (2011) 82:761–5. 10.1136/jnnp.2010.229740 21436230 PMC3696191

[58] D'Souza M Chen KS Rosenberg J Elias WJ Eisenberg HM Gwinn R. Impact of skull density ratio on efficacy and safety of magnetic resonance-guided focused ultrasound treatment of essential tremor. J Neurosurg. (2019) 132:1392–7. 10.3171/2019.2.JNS183517 31026836

[59] Boutet A Gwun D Gramer R Ranjan M Elias GJB Tilden D. The relevance of skull density ratio in selecting candidates for transcranial MR-guided focused ultrasound. J Neurosurg. (2019) 132:1785–91. 10.3171/2019.2.JNS182571 31051458

[60] Gallay MN Jeanmonod D Liu J Morel A. Human pallidothalamic and cerebellothalamic tracts: anatomical basis for functional stereotactic neurosurgery. Brain Struct Funct. (2008) 212:443–63. 10.1007/s00429-007-0170-0 18193279 PMC2494572

[61] Blomstedt P Sandvik U Fytagoridis A Tisch S. The posterior subthalamic area in the treatment of movement disorders: past, present, and future. Neurosurgery. (2009) 64:1029–38. Discussion 1038–1042. 10.1227/01.NEU.0000345643.69486.BC 19487881

[62] Sammartino F Krishna V King NK Lozano AM Schwartz ML Huang Y. Tractography-based ventral intermediate nucleus targeting: novel methodology and intraoperative validation. Mov Disord. (2016) 31:1217–25. 10.1002/mds.26633 27214406 PMC5089633

[63] Yamada K Akazawa K Yuen S Goto M Matsushima S Takahata A. MR imaging of ventral thalamic nuclei. AJNR Am J Neuroradiol. (2010) 31:732–5. 10.3174/ajnr.A1870 19926703 PMC7964215

[64] Soares JM Marques P Alves V Sousa N. A hitchhiker's guide to diffusion tensor imaging. Front Neurosci. (2013) 7:31. 10.3389/fnins.2013.00031 23486659 PMC3594764

[65] O'Donnell LJ Westin CF. An introduction to diffusion tensor image analysis. Neurosurg Clin N Am. (2011) 22:185–96. 10.1016/j.nec.2010.12.004 21435570 PMC3163395

