# Supplementary material for: Outcome and Complications of MR Guided Focused Ultrasound for Essential Tremor: A Systematic Review and Meta-Analysis
Source: Front Neurol. 2021 May 7;12:654711. doi: 10.3389/fneur.2021.654711 (PMC8137896; doi:10.3389/fneur.2021.654711)
Supplement: Supplementary file 1 [file Data_Sheet_1.docx]

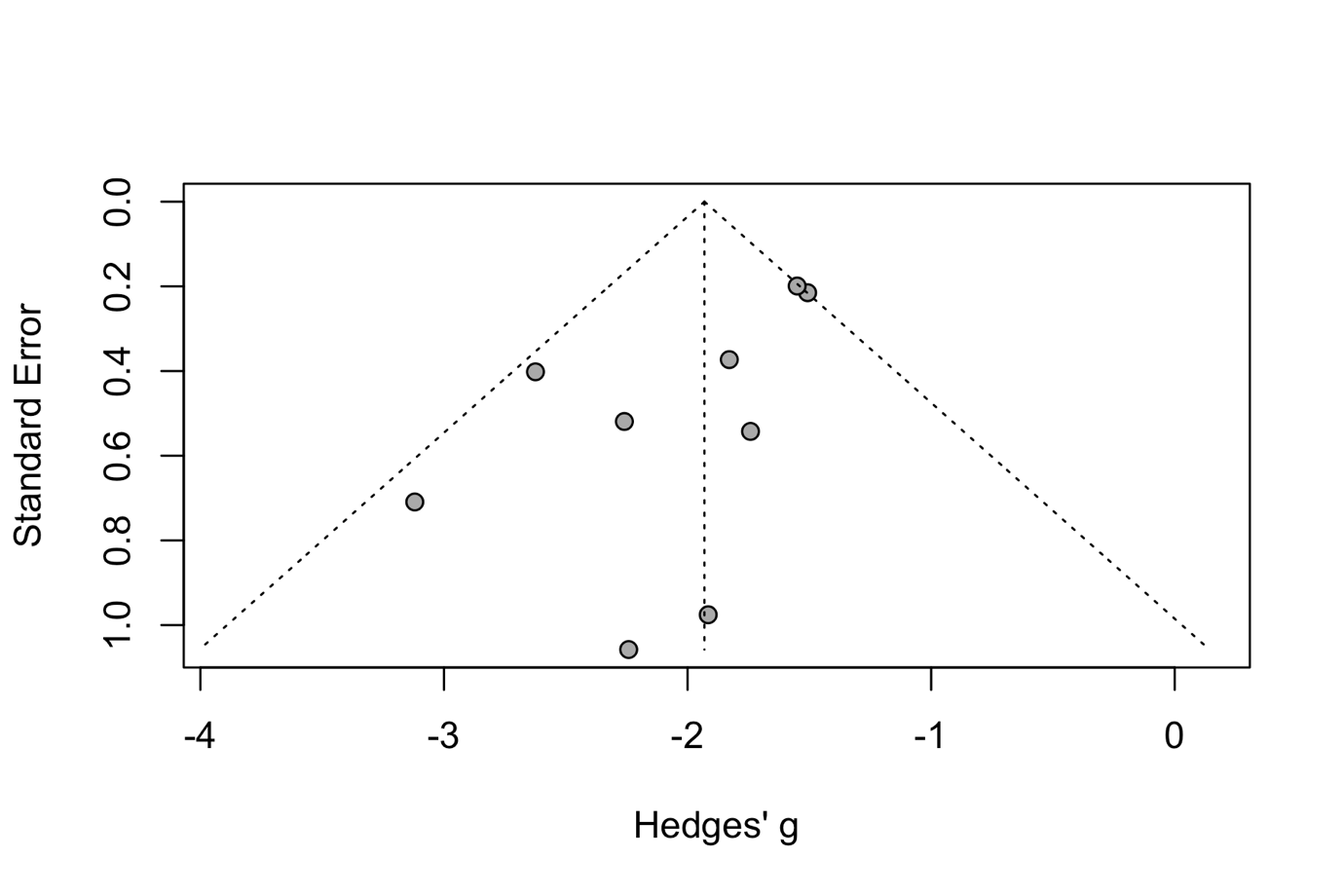


**SDC 1. Figure**: Funnel plot of studies reporting total CRST score shows a well distributed plot with no publication bias.
