# Supplementary material for: Outcome and Complications of MR Guided Focused Ultrasound for Essential Tremor: A Systematic Review and Meta-Analysis
Source: Front Neurol. 2021 May 7;12:654711. doi: 10.3389/fneur.2021.654711 (PMC8137896; doi:10.3389/fneur.2021.654711)
Supplement: Supplementary file 2 [file Data_Sheet_2.docx]

SDC 2. Table: Immediate Complications (during treatment until 48 hours).

Studies reporting zero complications are marked as ‘0’. Studies in which no complication data was reported for respective time period are marked as ‘NA’

Period in brackets denotes time until when the complication persisted.

(# complications not mentioned separately for ET patients)

| **Study** | **NEUROLOGICAL** | | | | | | | | | | | | | **MINOR/TREATMENT RELATED** | | | | | | | | |
| --- | --- | --- | --- | --- | --- | --- | --- | --- | --- | --- | --- | --- | --- | --- | --- | --- | --- | --- | --- | --- | --- | --- |
|  | **Sensory** | | | | | **Gait** | | | | **Motor** | **Speech & Swallowing** | | | **Headache & Fatigue** | | | **Sonication Related** | | **Frame & MRI Related** | | | **Other** |
|  | **Paresthesia** | **Taste Disturbance** | **Dysesthesia`** | **Tinnitus** | **TOTAL** | **Dizziness** | **Gait Ataxia** | **Dysmetria/Hand Ataxia** | **TOTAL** |  | **Slurred Speech** | **Dysphagia** | **TOTAL** | **Headache** | **Fatigue/Asthenia** | **TOTAL** | Sonification Related | **TOTAL** | **Frame Related** | **MRI Related** | **TOTAL** |  |
| Lipsman et al, March 2013[24] | 2 | 0 | 0 | 0 | **2** | 0 | 0 | 0 | **0** | **0** | 0 | 0 | **0** | 0 | 0 | **0** | 0 | **0** | 0 | 0 | **0** | **1 DVT** |
| Elias et al, August 2013[25] | 14 | 0 | 1 | 0 | **15** | 5 | 4 | 1 | **10** | **1 (Grip)** | 0 | 1 | **1** | 0 | 0 | **0** | Head Pain-9, Flushed sensation-4, Light headedness-6, Dizziness-5,Nausea-5, Emesis-3, Syncope-1 | **33** | Headache-4, Scalp numbness-4, Pin site laceration-1, Periorbital edema-1 | Scalp burn due to pinsite heating-2 | **12** | **0** |
| Chang et al, May 2014 [26] | 0 | 0 | 0 | 0 | **0** | 0 | 1 | 0 | **1** | **0** | 0 | 0 | **0** | 0 | 0 | **0** | Dizziness, Nausea & Vomiting-5 | **5** | 0 | 0 | **0** | **3 failed to attain temperature above 50 C** |
| Gallay et al, February 2016 [27] | 0 | 0 | 0 | 0 | **0** | 0 | 5 | 0 | **5** | **0** | 0 | 0 | **0** | 0 | 0 | **0** | 0 | **0** | 0 | 0 | **0** | **0** |
| Elias et al, August 2016 [28] (Treatment group) ^*^ | 21 | 3 | 0 | 3 | **27** | 5 | 20 (11 objective, 9 subjective) | 7 | **32** | **2 (Grip)** | 1 | 1 | **2** | 8 | 3 | **11** | Head Pain-17, Vertigo-12, Nausea-11,Vomiting-2, Scalp Tingling-4, Back pain-5, Anxiety-3 | **54** | Pin site pain/bruising - 17 | 0 | **17** | **0** |
| Elias et al, August 2016 [28] (Sham Crossover) ^*^ | 11 | 2 | 0 | 0 | **13** | 3 | 8 (3 objective, 5 subjective) | 3 | **14** | **3 (Grip)** | 0 | 3 | **3** | 7 | 4 | **11** | Head pain-7, Nausea-3, Vomiting-1, Dizzy-4, Vertigo-1 | **16** | Pin site pain/bruising - 7 | 0 | **7** | **0** |
| Chang et al, December 2017[29] | NA | NA | NA | NA | **NA** | NA | NA | NA | **NA** | **NA** | NA | NA | **NA** | NA | NA | **NA** | NA | **NA** | NA | NA | **NA** | **NA** |
| Halpern et al, November 2019 [30] | NA | NA | NA | NA | **NA** | NA | NA | NA | **NA** | **NA** | NA | NA | **NA** | NA | NA | **NA** | NA | **NA** | NA | NA | **NA** | **NA** |
| Zaroor et al, February 2017 [31] | 0 | 4 | 0 | 0 | **4** | 4 | 3 | 2 | **9** | **0** | 0 | 0 | **0** | 0 | 4 | **4** | Vertigo-14, Head pain-11, Dizziness-4, Nausea-3, Burning skull sensation-3, vomiting-2, lip paresthesia-2 | **39** | Scalp numbness - 5, pin site hematoma - 3 | 0 | **8** | **0** |
| Schreglmann et al, March 2017 [32] | 0 | 0 | 0 | 0 | **0** | 0 | 2 (1 objective, 1 subjective) | 1 | **3** | **0** | 0 | 0 | **0** | 0 | 0 | **0** | Dizziness-4 | **4** | 0 | 0 | **0** | **0** |
| Kim et al, August 2017[33] | 0 | 1 | 0 | 0 | **1** | 0 | 1 | 0 | **1** | **2 (Facial)** | 0 | 0 | **0** | 0 | 0 | **0** | 0 | **0** | 0 | 0 | **0** | **0** |
| Chazen et al, October 2017 [34] | NA | NA | NA | NA | **NA** | NA | NA | NA | **NA** | **NA** | NA | NA | **NA** | NA | NA | **NA** | NA | **NA** | NA | NA | **NA** | **NA** |
| Federau et al, October 2017[35] | NA | NA | NA | NA | **NA** | NA | NA | NA | **NA** | **NA** | NA | NA | **NA** | NA | NA | **NA** | NA | **NA** | NA | NA | **NA** | **NA** |
| Jung et al, February 2018[36] | 0 | 0 | 0 | 0 | **0** | 0 | 1 | 0 | **1** | **0** | 0 | 0 | **0** | 0 | 0 | **0** | Headache, Dizziness or nausea - 10 | **10** | Pin site pain-3 | 0 | **3** | **0** |
| Iacopino et al, February 2018[37] | 2 | 0 | 0 | 0 | **2** | 0 | 4 objective (2 disappeared within 48 hours), 2 subjective | 0 | **6** | **1 (Grip)** | 0 | 0 | **0** | 0 | Most (not quantified) | **0** | Dizzy-4, Vertigo-4, Postural instability-1, Tinnitus - 1 (water flushing around head) | **10 - not mentioned separately for ET** | Headache-3, scalp numbness - 1, laceration pin site-1, frame displacement - 1 | 0 | **6 - not mentioned separately for ET** | **4 ET patients - aborted treatment due to severe headache; 1 ET patient - failed to attain ablative temperature** |
| Krishna et al, March 2018[38] | 0 | 0 | 0 | 0 | **0** | 0 | 3 objective, 1 balance difficulty | 0 | **4** | **0** | 0 | 0 | **0** | 0 | 0 | **0** | 0 | **0** | 0 | 0 | **0** | **0** |
| Boutet et al, November 2018[39] | 12 | 0 | 0 | 0 | **12** | 0 | 41 | 21 | **62** | **13** | 0 | 3 | **3** | 0 | 0 | **0** | NA | **NA** | NA | NA | **NA** | **0** |
| Park et al, February 2019[40] | 1 | 0 | 0 | 0 | **1** | 0 | 1 | 0 | **1** | **0** | 0 | 0 | **0** | 0 | 0 | **0** | Dizziness - 1, Nausea and vomiting - 1 | **2** | 0 | 0 | **0** | **0** |
| Hori et al, February 2019[41] | NA | NA | NA | NA | **NA** | NA | NA | NA | **NA** | **NA** | NA | NA | **NA** | NA | NA | **NA** | NA | **NA** | NA | NA | **NA** | **NA** |
| Pineda-Pardo et al, March 2019[42] | 4 | 0 | 0 | 0 | **4** | 0 | 1 objective, 6 subjective, | 0 | **7** | **0** | 0 | 1 | **1** | 0 | 0 | **0** | 0 | **0** | 0 | 0 | **0** | **0** |
| Yang et al, March 2019[43] | 0 | 0 | 0 | 0 | **0** | 0 | 0 | 0 | **0** | **0** | 0 | 0 | **0** | 0 | 0 | **0** | 0 | **0** | 0 | 0 | **0** | **0** |
| Jones et al, May 2019[44] | NA | NA | NA | NA | **NA** | NA | NA | NA | **NA** | **NA** | NA | NA | **NA** | NA | NA | **NA** | NA | **NA** | NA | NA | **NA** | **NA** |
| Sinai et al, July 2019[45] | 6 | 5 | 0 | 0 | **11** | 0 | 10 objective, 8 subjective | 6 | **24** | **0** | 0 | 0 | **0** | 0 | 4 | **4** | Vertigo-23, Headache-17, Nausea-6, Head/scalp heat-5, Vomiting-2, Dizzy-2, Paresthesia-10 | **65** | Scalp numbness-1 | 0 | **1** | **0** |
| Chang et al, July 2019[46] | NA | NA | NA | NA | **NA** | NA | NA | NA | **NA** | **NA** | NA | NA | **NA** | NA | NA | **NA** | NA | **NA** | NA | NA | **NA** | **NA** |
| Miller et al, August 2019[47] | NA | NA | NA | NA | **NA** | NA | NA | NA | **NA** | **NA** | NA | NA | **NA** | NA | NA | **NA** | NA | **NA** | NA | NA | **NA** | **NA** |
| Krishna et al, November 2019 [48] (Pivotal) ^*^ | NA | NA | NA | NA | **NA** | NA | NA | NA | **NA** | **NA** | NA | NA | **NA** | NA | NA | **NA** | NA | **NA** | NA | NA | **NA** | **NA** |
| Krishna et al, November 2019 [48] (Post Pivotal) ^*^ | NA | NA | NA | NA | **NA** | NA | NA | NA | **NA** | **NA** | NA | NA | **NA** | NA | NA | **NA** | NA | **NA** | NA | NA | **NA** | **NA** |
| Gallay et al, February 2020[49] | 1 | 0 | 0 | 0 | **1** | 0 | 2 (objective), 5 (subjective) | 0 | **7** | **0** | 0 | 5 | **5** | 0 | 0 | **0** | Head Pain - 4 | **4** | Frontal scalp swelling - 1 | 0 | **1** | **0** |
| Paff et al, March 2020[50] | 0 | 0 | 0 | 0 | **0** | 0 | 1 | 0 | **1** | **1 (c/l lower limb)** | 0 | 0 | **0** | 0 | 0 | **0** | 0 | **0** | 0 | 0 | **0** | **0** |
| Buch et al, May 2020 [51] | NA | NA | NA | NA | **NA** | NA | NA | NA | **NA** | **NA** | NA | NA | **NA** | NA | NA | **NA** | NA | **NA** | NA | NA | **NA** | **NA** |
| Fukutome et al, May 2020 [52] | 1 | 0 | 0 | 0 | **1** | 0 | 1 | 0 | **1** | **0** | 0 | 0 | **0** | 0 | 0 | **0** | Headache, Vomiting or Dizziness - 9 | **9** | 0 | 0 | **0** | **0** |
