# Supplementary material for: Outcome and Complications of MR Guided Focused Ultrasound for Essential Tremor: A Systematic Review and Meta-Analysis
Source: Front Neurol. 2021 May 7;12:654711. doi: 10.3389/fneur.2021.654711 (PMC8137896; doi:10.3389/fneur.2021.654711)
Supplement: Supplementary file 3 [file Data_Sheet_3.docx]

SDC 3. Table: Short Term Complications (2 days post procedure to 3 months).

Studies reporting zero complications are marked as ‘0’. Studies in which no complication data was reported for respective time period are marked as ‘NA’

Period in brackets denotes time until when the complication persisted.

(# complications not mentioned separately for ET patients)

| **Study** | **NEUROLOGICAL** | | | | | | | | | | | | | **MINOR/TREATEMENT RELATED** | | | | | | | |
| --- | --- | --- | --- | --- | --- | --- | --- | --- | --- | --- | --- | --- | --- | --- | --- | --- | --- | --- | --- | --- | --- |
|  | **Sensory** | | | | | **Gait** | | | | **Motor** | **Speech & Swallowing** | | | **Headache & Fatigue** | | | **Sonication Related** | **Frame & MRI Related** | | | **Other** |
|  | **Paresthesia** | **Taste Disturbance** | **Dysesthesia`** | **Tinnitus** | **TOTAL** | **Dizziness** | **Gait Ataxia** | **Dysmetria/Hand Ataxia** | **TOTAL** |  | **Slurred Speech** | **Dysphagia** | **TOTAL** | **Headache** | **Fatigue/Asthenia** | **TOTAL** |  | **Frame Related** | **MRI Related** | **TOTAL** |  |
| Lipsman et al, March 2013[24] | 1 | 0 | 0 | 0 | **1** | 0 | 0 | 0 | **0** | **0** | 0 | 0 | **0** | 0 | 0 | **0** | **0** | 0 | 0 | **0** | **1 DVT** |
| Elias et al, August 2013[25] | 3 | 0 | 1 | 0 | **4** | 0 | 4 (<1m) | 1 (< 1m) | **5** | **1 (5 days)** | 0 | 0 | **0** | 0 | 0 | **0** | **0** | 0 | 0 | **0** | **0** |
| Chang et al, May 2014 [26] | 0 | 0 | 0 | 0 | **0** | 0 | 1 (1m) | 0 | **1** | **0** | 0 | 0 | **0** | 0 | 0 | **0** | **0** | 0 | 0 | **0** | **0** |
| Gallay et al, February 2016 [27] | 0 | 0 | 0 | 0 | **0** | 0 | 5 | 0 | **5** | **0** | 0 | 0 | **0** | 0 | 0 | **0** | **0** | 0 | 0 | **0** | **0** |
| Elias et al, August 2016 [28] (Treatment group) ^*^ | 14 | 2 | 0 | 1(1m) | **17** | 3 | 9 (2 objective, 7 subjective) | 5 | **17** | **2 (Grip)** | 1 | 1 | **2** | 2 | 1 | **3** | **0** | 0 | 0 | **0** | **0** |
| Elias et al, August 2016 [28] (Sham Crossover) ^*^ | 6 | 2 | 0 | 0 | **8** | 1 | 4 (2 objective, 2 subjective) | 2 | **7** | **2 (Grip)** | 0 | 2 | **2** | 0 | 2 | **2** | **0** | 0 | 0 | **0** | **0** |
| Chang et al, December 2017[29] | NA | NA | NA | NA | **NA** | NA | NA | NA | **NA** | **NA** | NA | NA | **NA** | NA | NA | **NA** | **NA** | NA | NA | **NA** | **NA** |
| Halpern et al, November 2019 [30] | NA | NA | NA | NA | **NA** | NA | NA | NA | **NA** | **NA** | NA | NA | **NA** | NA | NA | **NA** | **NA** | NA | NA | **NA** | **NA** |
| Zaroor et al, February 2017 [31] | 0 | 4 (1-3m) | 0 | 0 | **4** | 4 (1-4weeks) | 3 (1-3m) | 2 (1-4weeks) | **9** | **0** | 0 | 0 | **0** | 0 | 0 | **0** | **0** | Scalp numbness-5 (1-4weeks), Pinsite hematoma-3(1-2weeks) | 0 | **8** | **0** |
| Schreglmann et al, March 2017 [32] | 0 | 0 | 0 | 0 | **0** | 0 | 2 (resolved within 3m) | 1 (resolved within 3m) | **3** | **0** | 0 | 0 | **0** | 0 | 0 | **0** | **0** | 0 | 0 | **0** | **0** |
| Kim et al, August 2017[33] | 0 | 1 (1 week) | 0 | 0 | **1** | 0 | 1 (1m) | 0 | **1** | **2 (Facial- 1 resolved in 1 month)** | 0 | 0 | **0** | 0 | 0 | **0** | **0** | 0 | 0 | **0** | **0** |
| Chazen et al, October 2017 [34] | NA | NA | NA | NA | **NA** | NA | NA | NA | **NA** | **NA** | NA | NA | **NA** | NA | NA | **NA** | **NA** | NA | NA | **NA** | **NA** |
| Federau et al, October 2017[35] | NA | NA | NA | NA | **NA** | NA | NA | NA | **NA** | **NA** | NA | NA | **NA** | NA | NA | **NA** | **NA** | NA | NA | **NA** | **NA** |
| Jung et al, February 2018[36] | 0 | 0 | 0 | 0 | **0** | 0 | 1 (1m) | 0 | **1** | **0** | 0 | 0 | **0** | 0 | 0 | **0** | **0** | 0 | 0 | **0** | **0** |
| Iacopino et al, February 2018[37] | 0 | 0 | 0 | 0 | **0** | 0 | 2 objective (1 - resolved within 3m), 1 subjective | 0 | **3** | **1 (Grip - 1 week)** | 0 | 0 | **0** | 0 | 0 | **0** | **0** | 0 | 0 | **0** | **0** |
| Krishna et al, March 2018[38] | 0 | 1 | 0 | 0 | **1** | 0 | 3(2-1m, 1-3m) | 0 | **3** | **0** | 0 | 0 | **0** | 1 | 0 | **0** | **0** | 0 | 0 | **0** | **0** |
| Boutet et al, November 2018[39] | 5 | 0 | 0 | 0 | **5** | 0 | 11 | 9 | **20** | **6** | 0 | 3 | **3** | 0 | 0 | **0** | **NA** | NA | NA | **NA** | **0** |
| Park et al, February 2019[40] | 1 (3m) | 0 | 0 | 0 | **1** | 0 | 1 | 0 | **1** | **0** | 0 | 0 | **0** | 0 | 0 | **0** | **0** | 0 | 0 | **0** | **0** |
| Hori et al, February 2019[41] | NA | NA | NA | NA | **NA** | NA | NA | NA | **NA** | **NA** | NA | NA | **NA** | NA | NA | **NA** | **NA** | NA | NA | **NA** | **NA** |
| Pineda-Pardo et al, March 2019[42] | 4 | 0 | 0 | 0 | **4** | 0 | 6 - subjective (3m), 1 objective | 0 | **7** | **0** | 0 | 1 | **1** | 0 | 0 | **0** | **0** | 0 | 0 | **0** | **0** |
| Yang et al, March 2019[43] | 0 | 0 | 0 | 0 | **0** | 0 | 0 | 0 | **0** | **0** | 0 | 0 | **0** | 0 | 0 | **0** | **0** | 0 | 0 | **0** | **0** |
| Jones et al, May 2019[44] | NA | NA | NA | NA | **NA** | NA | NA | NA | **NA** | **NA** | NA | NA | **NA** | NA | NA | **NA** | **NA** | NA | NA | **NA** | **NA** |
| Sinai et al, July 2019[45] | 6 (1 resolved over 1m, 2 resolved over 3m) | 5 (3 resolved over 3m) | 0 | 0 | **11** | 0 | 10 (8 resolved over 1-3m), 8 subjective unsteadiness resolved over 1 week | 6 (1m) | **24** | **0** | 0 | 0 | **0** | 0 | 4 (1m) | **4** | **0** | Scalp numbness - 1 (3m) | 0 | **1** | **0** |
| Chang et al, July 2019[46] | NA | NA | NA | NA | **NA** | NA | NA | NA | **NA** | **NA** | NA | NA | **NA** | NA | NA | **NA** | **NA** | NA | NA | **NA** | **NA** |
| Miller et al, August 2019[47] | NA | NA | NA | NA | **NA** | NA | NA | NA | **NA** | **NA** | NA | NA | **NA** | NA | NA | **NA** | **NA** | NA | NA | **NA** | **NA** |
| Krishna et al, November 2019 [48] (Pivotal) ^*^ | NA | NA | NA | NA | **NA** | NA | NA | NA | **NA** | **NA** | NA | NA | **NA** | NA | NA | **NA** | **NA** | NA | NA | **NA** | **NA** |
| Krishna et al, November 2019 [48] (Post Pivotal) ^*^ | NA | NA | NA | NA | **NA** | NA | NA | NA | **NA** | **NA** | NA | NA | **NA** | NA | NA | **NA** | **NA** | NA | NA | **NA** | **NA** |
| Gallay et al, February 2020[49] | 1 | 0 | 0 | 0 | **1** | 0 | 3 objective, 2 subjective | 0 | **5** | **0** | 0 | 1 | **1** | 0 | 0 | **0** | **0** | 1 scalp swelling (few days) | 0 | **1** | **0** |
| Paff et al, March 2020[50] | 0 | 0 | 0 | 0 | **0** | 0 | 1 (1m) | 0 | **1** | **1 (c/l lower limb) (1m)** | 0 | 0 | **0** | 0 | 0 | **0** | **0** | 0 | 0 | **0** | **0** |
| Buch et al, May 2020 [51] | NA | NA | NA | NA | **NA** | NA | NA | NA | **NA** | **NA** | NA | NA | **NA** | NA | NA | **NA** | **NA** | NA | NA | **NA** | **NA** |
| Fukutome et al, May 2020 [52] | 1 | 0 | 0 | 0 | **1** | 0 | 1 | 0 | **1** | **0** | 0 | 0 | **0** | 0 | 0 | **0** | **0** | 0 | 0 | **0** | **0** |
