# Supplementary material for: Outcome and Complications of MR Guided Focused Ultrasound for Essential Tremor: A Systematic Review and Meta-Analysis
Source: Front Neurol. 2021 May 7;12:654711. doi: 10.3389/fneur.2021.654711 (PMC8137896; doi:10.3389/fneur.2021.654711)
Supplement: Supplementary file 5 [file Data_Sheet_5.docx]

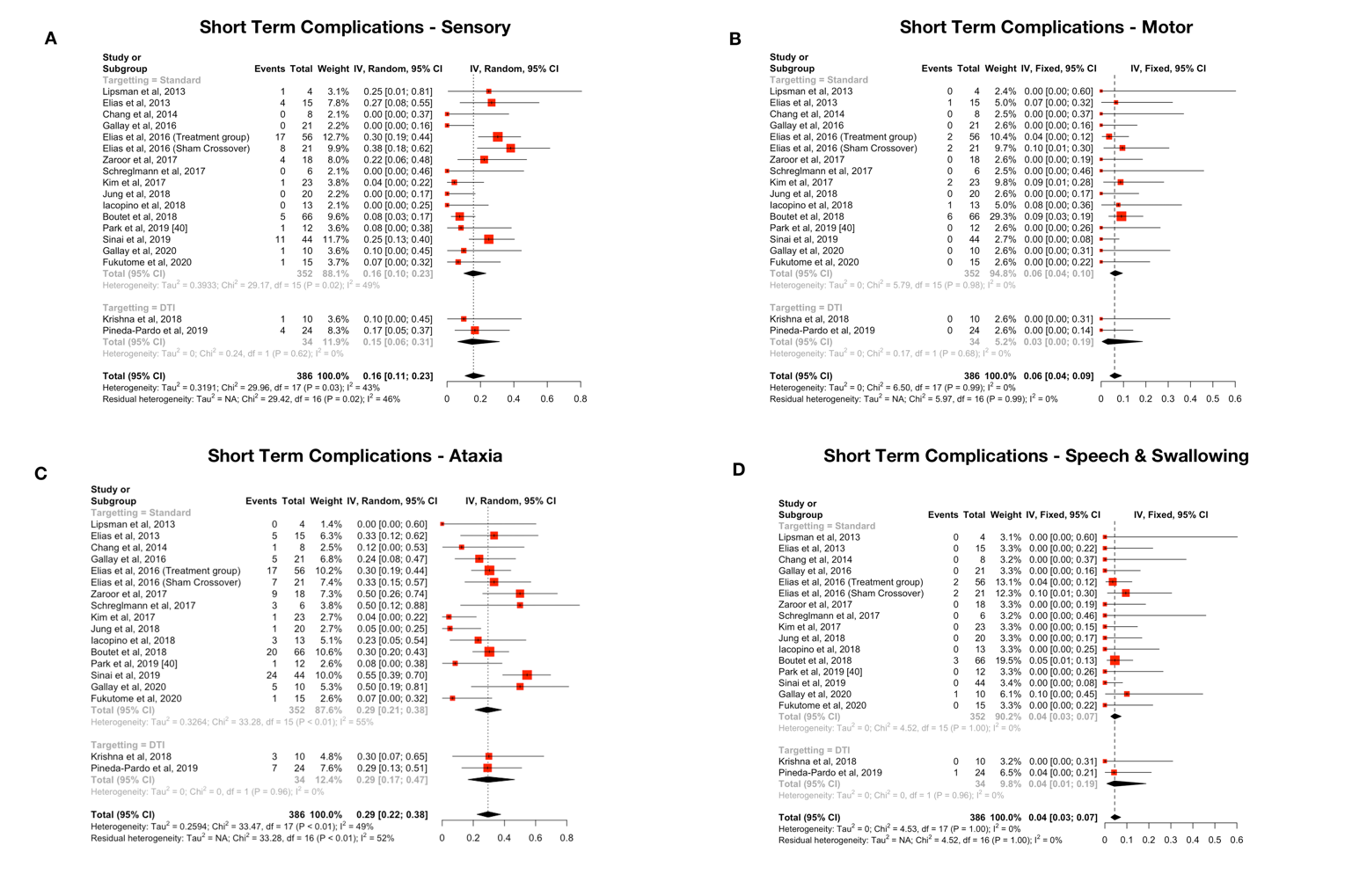


**SDC 5. Figure 1**: Forest plots depicting pooled proportions of short term sensory (A), motor (B), ataxia (C) and speech & swallowing (D) related complications.


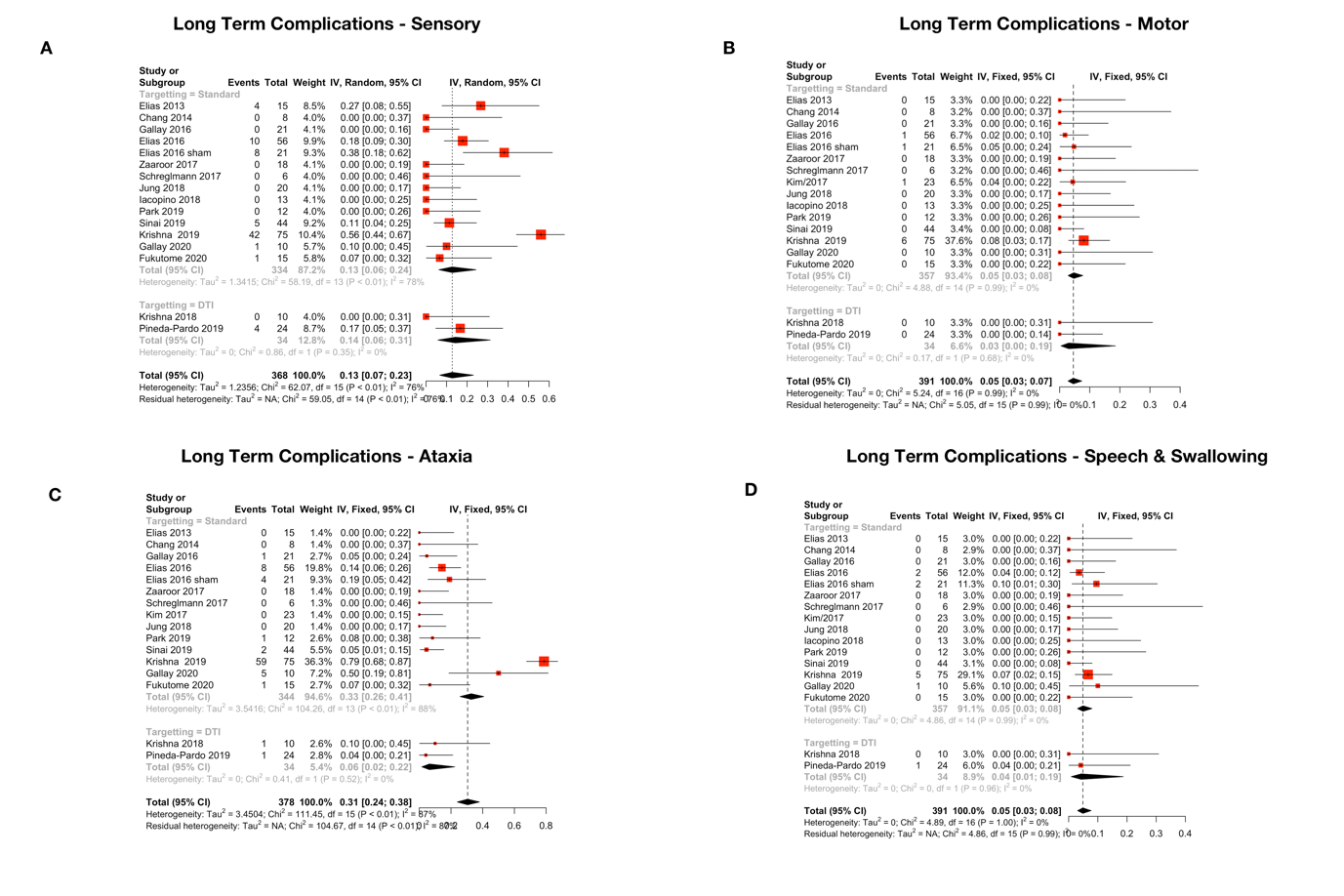


**SDC 5. Figure 2**: Forest plots depicting pooled proportions of long term sensory (A), motor (B), ataxia (C) and speech & swallowing (D) related complications.
